# Carbon-to-ATP ratios across the kingdoms of life

**DOI:** 10.1016/j.isci.2026.116849

**Published:** 2026-07-29

**Authors:** Peyman Fahimi, Mohammad M. Amirian, Andrew J. Irwin, Zoe V. Finkel

**Affiliations:** 1 Department of Mathematics & Statistics, Dalhousie University, Halifax, NS B3H4R2, Canada; 2 Department of Oceanography, Dalhousie University, Halifax, NS B3H4R2, Canada

**Keywords:** carbon-to-ATP ratio, marine microbial biomass, respiration rate, ATP turnover time, structural materials

## Abstract

Estimating biomass is essential for quantifying energy flow, nutrient cycling, and ecosystem functioning across the biosphere. For nearly 60 years, the cellular carbon-to-ATP ratio (C:ATP, g/g) has been used to estimate marine microbial biomass, often assuming a value near 250. Here we compile ∼400 measurements from more than 80 studies spanning bacteria, unicellular eukaryotes, animals, plants, and tissues, and show that C:ATP varies by five orders of magnitude across species, physiological states, and environmental conditions. We then develop a mechanistic model demonstrating this variation arises from differences in ATP production rate, ATP turnover time, and the fraction of metabolically active carbon. The model predicts that median C:ATP is within a factor of two of 250 for bacteria and unicellular marine eukaryotes, while systematically differing in other groups. These results indicate that C:ATP should be treated as a context-dependent physiological quantity in biomass estimation and metabolic modeling across organisms and environments.

## Highlights

• C:ATP links biomass estimation to organismal energy metabolism
• C:ATP varies by five orders of magnitude across species and conditions
• ATP production, turnover time, and functional carbon explain C:ATP variation
• C:ATP should be treated as a context-dependent physiological trait

## Graphical abstract

**Figure:**
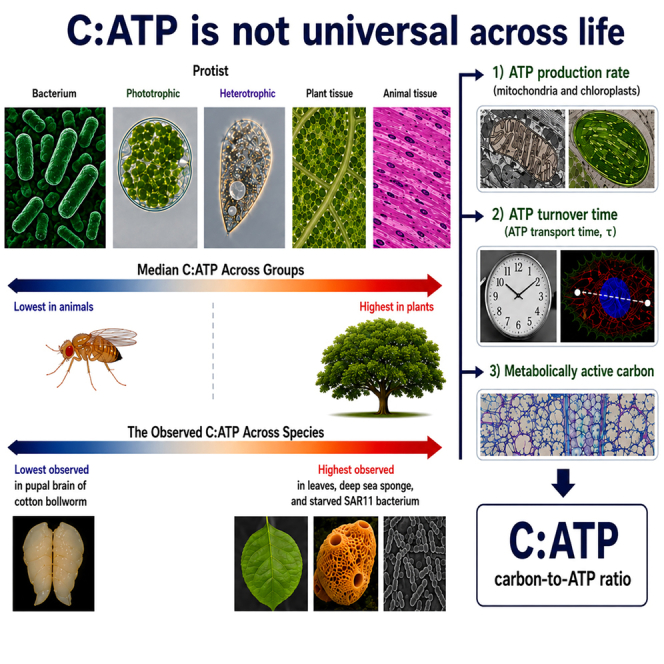

## Teaser

Ecology; Biological sciences

## Introduction

Understanding the energetic costs of building and operating living systems is essential to explaining patterns of growth, development, and ecological trade-offs across species.^1^^,^^2^ These costs, measured in terms of ATP (adenosine 5′-triphosphate) molecules that fuel most cellular processes,^3^ are fundamental for understanding how organisms allocate resources and function. A central question in bioenergetics is how much ATP mass is available per unit of metabolically active carbon, expressed as the C:ATP mass ratio. Beyond its fundamental significance, this ratio has long been used for microbial biomass estimation.

Researchers collect water-column samples, measure particulate carbon and ATP, and then apply the long-standing benchmark of C:ATP (g/g) = 250:1 to estimate the fraction of particulate carbon associated with living microbial biomass versus nonliving detrital material at each depth. In other words, deviations from this benchmark are typically attributed to detritus, dead cells, or methodological differences.^4^^,^^5^^,^^6^^,^^7^ However, these samples originate from physiologically and environmentally diverse regions with varying organismal composition, where conditions change dynamically. To challenge the assumption of a constant C:ATP ratio, we compiled approximately 400 data points from numerous independent studies published over the past 60 years (see Data S1) showing that this ratio varies widely across species (highest in leaves, deep sea sponge, and starved SAR11, lowest in the pupal brain of the cotton bollworm), physiological states (e.g., organism size, growth rate, carbon source, workload, stress exposure, developmental stage), and environmental conditions (e.g., nutrient and oxygen availability, temperature, salinity). We seek the physiological meaning behind this ratio and argue that such variability cannot be dismissed as experimental artifacts^5^^,^^8^ but should be understood mechanistically.

Because our compiled data reveal a statistically significant relationship between C:ATP and organism or tissue size (see compiled measurements of ATP content per unit biomass across the kingdoms of life, Figure S2; Table S1), we developed a size-dependent model of ATP and carbon content. Cellular ATP content ($\text{ATP},\frac{\text{gram}}{\text{organism}}$) can be computed as the product of ATP consumption rate and the average time between ATP production and consumption, known as the turnover time (*τ*, s).^9^ Under steady-state conditions in actively growing cells, the ATP production rate ($J_{\text{ATP}},\frac{\text{gram}}{\text{organism}\times s}$) approximately matches the ATP consumption rate.^9^^,^^10^ Therefore, ATP content can be expressed as the product of the ATP production rate and ATP turnover time (Equation 1), linking ATP content to measurable parameters including respiration and photosynthesis rates,^11^^,^^12^^,^^13^^,^^14^^,^^15^ as well as biophysical constraints such as ATP transport time.

(Equation 1) $$\text{ATP}=J_{\text{ATP}}\times \tau .$$

Although the links between respiration, photosynthesis, and ATP production rates are well established across taxa,^16^^,^^17^ and both respiration and photosynthesis scale with organism size,^1^^,^^18^ ATP turnover time remains poorly characterized, particularly in animals. Standard formulas for free diffusion and diffusive target search provide lower and upper bounds for size-dependent ATP turnover time, yet a more precise model would require detailed information on the copy number of mitochondria relative to cell volume, their spatial distribution within the 3D cellular space, the ATP consumption rate of different organelle types, and the packing density of cellular structures. These factors collectively determine how ATP is transported from mitochondria to sites of consumption, and thus the true ATP turnover time. Developing such a comprehensive model is beyond the scope of this paper. Instead, we address the ATP turnover time problem by combining empirical ATP content data with theoretical estimates of ATP production rates to infer turnover times, and then examine their biophysical underpinnings through cell size, mitochondrial positioning, and mitochondrial abundance. Carbon content is strongly size-dependent and considerably more stable than ATP content, yet it varies with structural features such as cell walls and tissue type. By integrating these elements, we construct a mechanistic model that not only explains the median C:ATP ratio but also accounts for its variation within and across kingdoms as a universal metric.

## Results

ATP and carbon content can be modeled in heterotrophs and photoautotrophs, from bacteria to animals and plants, as the outcome of interactions among underlying biological, biochemical, and biophysical variables. Herein we design such a model and test it against an extensive database compiled from 60 years of literature across the kingdoms of life. In summary, at steady state, organismal ATP content is expressed as the product of the ATP production rate and the ATP turnover time, ATP = *J*_ATP_×*τ*, where *J*_ATP_ is calculated from respiration in heterotrophs and from the combined contributions of respiration and photosynthesis in photoautotrophs, accounting for proton leak and ATP/O_2_ stoichiometry, and *τ* (s) represents the average time between ATP production and consumption, including intracellular transport. Carbon content is estimated independently from organism dry mass using empirical scaling relationships, with structural material (SM) treated as a variable fraction of dry mass that may be metabolically inactive and is therefore partially excluded. The model integrates these components using literature-driven scaling laws for respiration rate, photosynthesis rate, carbon content, and SM, and is evaluated against a compiled dataset of ∼400 measurements of ATP and carbon content across taxa and tissues (see also compiled measurements of ATP content per unit biomass across the kingdoms of life and data compilation from the literature for the theoretical model).

### ATP content

In aerobic heterotrophs, the majority of ATP (∼90%) is generated via oxidative phosphorylation, with the remainder produced through substrate-level phosphorylation in glycolysis and the small GTP yield of the tricarboxylic acid (TCA) cycle.^17^ Within the mitochondrial matrix, NADH and FADH_2_ donate electrons to the electron transport chain, ultimately reducing oxygen to water. The released energy pumps protons across the membrane, and their return through ATP synthase drives ATP synthesis (with bacteria using an analogous chemiosmotic system operating across their cytoplasmic membrane).^3^^,^^19^^,^^20^ A portion of protons bypasses ATP synthase, re-entering the mitochondrial matrix without contributing to ATP synthesis, thereby dissipating energy as heat.^21^ This phenomenon, known as proton leak (*PL*_1_), arises from various factors, including the high proton concentration in the intermembrane space, intrinsic properties of the phospholipid membrane, and the presence of water wires and anion carrier proteins^22^ (see proton leak in mitochondria and bacteria for factors affecting proton leak and the corresponding value of the leak). The stoichiometric ratio of oxygen consumption to ATP synthesis ( $\frac{\text{ATP}}{O_{2}^{c}}$; see stoichiometric ratios of ATP/O2 in oxidative phosphorylation)^17^ combined with the respiration rate ($RR,\frac{\text{mol}\;O_{2}}{\text{organism}}/s$) can be used to estimate the rate of ATP production. A proportion of respiration is lost due to proton leak, leaving *RR*(1-*PL*_1_) as the effective respiration rate contributing to ATP production. A substantial body of literature demonstrates that respiration rate varies among species within major taxonomic groups as a function of organism size^13^^,^^18^ and growth rate,^23^^,^^24^^,^^25^^,^^26^ with further variation observed across taxonomic groups due to factors such as gene number, genome length, surface area limitations, and circulatory system differences.^18^ We compiled data on heterotrophic and photoautotrophic organisms, from bacteria to mammals, to document the variation in respiration rate within and across taxonomic groups (see data compilation from the literature for the theoretical model). Accordingly, the ATP production rate in heterotrophs can be expressed by the following equation, where the constant 507.2 represents the molar mass of ATP:

(Equation 2) $${J_{\text{ATP}}}_{\text{Heterotrophs}}=RR\left(1-{PL}_{1}\right)\left(\frac{\text{ATP}}{O_{2}^{c}}\right)\left(507.2\right).$$

In photoautotrophs, additional processes (non-cyclic, cyclic, and pseudo-cyclic electron transport, see photosynthesis rate, proton leak, and ATPO2p ratio in chloroplast) produce ATP.^16^^,^^27^ Non-cyclic photophosphorylation in the chloroplast during photosynthesis involves a tightly regulated coupling between several key processes: the number of protons pumped into the thylakoid lumen, the number of oxygen molecules produced ($O_{2}^{p}$), and the number of ATP molecules generated and required for carbon fixation.^16^^,^^27^ The proportion of the total cellular ATP derived from photosynthesis versus respiration can be estimated using the dark respiration-to-photosynthesis ratio $\left(\frac{DRR}{\mathrm{PR}}\right)$,^28^^,^^29^ which varies both within and across species (see photosynthesis rate, proton leak, and ATPO2p ratio in chloroplast). Protons leak across the thylakoid membrane (PL_2_), as also observed across the mitochondrial membrane. Assuming the dark respiration rate (*DRR*) of photoautotrophic organisms reported in Table S6 is equivalent to their light respiration rate, the total ATP production rate in these organisms can be determined as follows:

(Equation 3) $${J_{\text{ATP}}}_{\text{Photoautotrophs}}=\;DRR\left(\left(1-{PL}_{1}\right)\frac{\text{ATP}}{O_{2}^{c}}+\frac{1}{\left(\frac{DRR}{\mathrm{PR}}\right)}\left(1-{PL}_{2}\right)\frac{\text{ATP}}{O_{2}^{p}}\right)\left(507.2\right).$$

ATP turnover time (*τ* in Equation 1) is the elapsed time from production to the consumption of an ATP molecule averaged over the entire organism. If ATP is not readily available at the site of consumption, its turnover time will include the time required for transport from the site of production. Therefore, it is crucial to know the position of mitochondria and their distance from the sites of ATP consumption. ATP turnover time has been measured and reported only in a limited number of studies—primarily in bacteria,^9^^,^^30^ microbial fungi,^30^ ciliates,^31^ and plants^30^^,^^32^—highlighting the scarcity of available data, particularly for animals where such measurements remain largely unexplored. ATP turnover time can be measured experimentally; for example, using ^32P^ radiolabeled phosphate tracer methods^33^ or by tracking the ATP decline during the initial second of aminoethyldextrane (AED) stimulation.^31^

**Figure 1:**
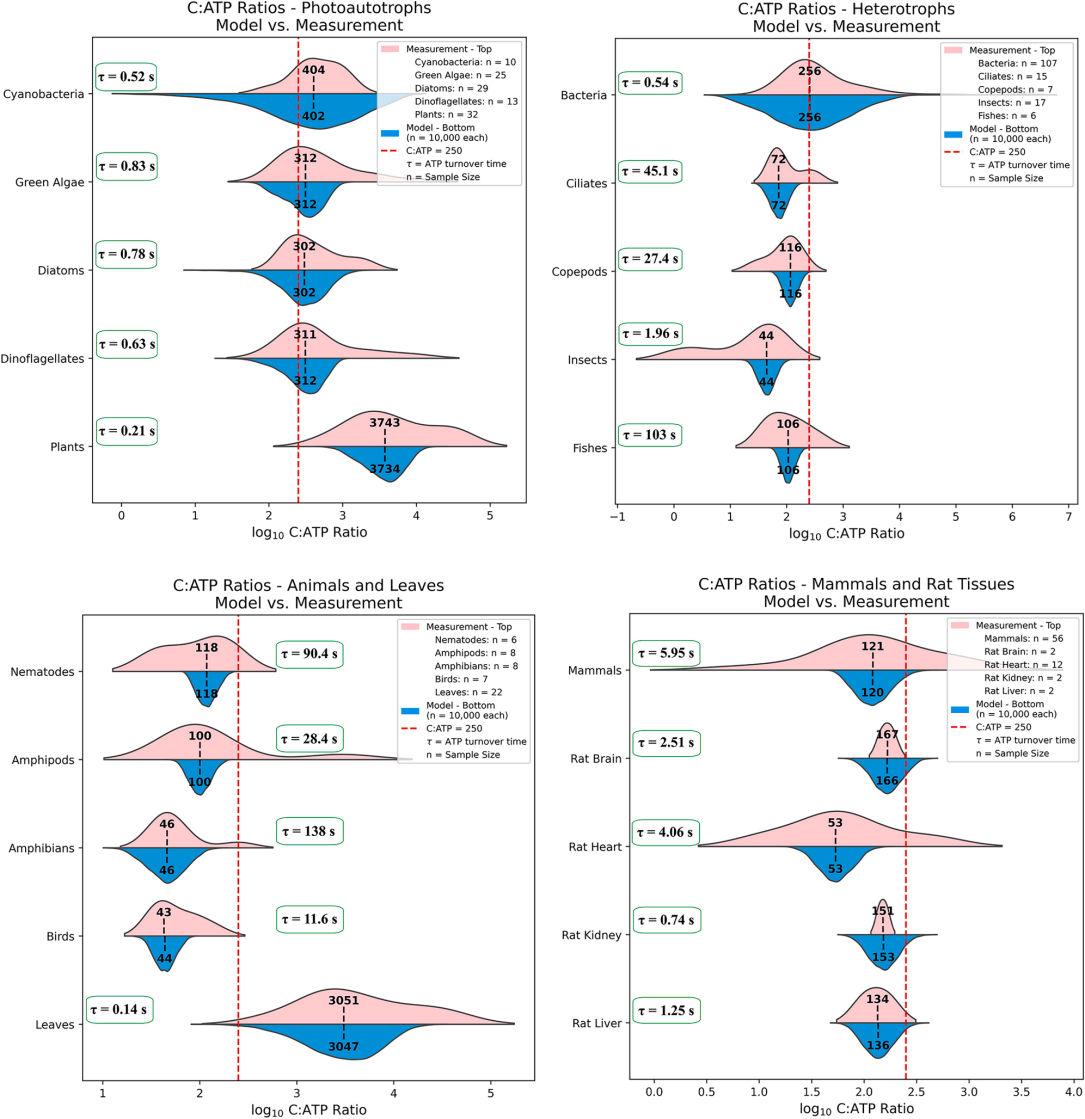
Comparison between experimental measurements and our theoretical steady-state model of the C:ATP ratio across major taxonomic groups, including unicellular and multicellular heterotrophs as well as photoautotrophs For cyanobacteria, both data and model include N_2_-fixing and non-N_2_-fixing strains. Plant experimental ATP values are primarily from leaves, with some data from roots, shoots, and plant cell lines, whereas model values represent greenhouse-grown tree seedlings. For bacteria, both experimental and modeled values reflect fast-growing strains except for SAR11 in the data. Experimental data for ciliates represent tintinnids and Paramecium, whereas the model encompasses a broader range of ciliate types. Insects are represented by relatively large terrestrial species in both the data and the model; experimental ATP values are mostly from flight muscle, with some from brain and fat body. For fishes, ATP measurements are from white muscle and sperm cells. ATP data for amphibians are taken from sartorius muscle, gastrocnemius, and brain of frogs. ATP data for birds are taken from pectoral muscle of pigeons, starlings, domestic fowl, and pheasants, and from erythrocytes of chicken, pigeon, and turkey. ATP data for leaves represent various species, while the model represents lettuce. Mammalian ATP data are from a mix of tissues—including heart, brain, muscle, liver, kidney, thigh, fat pad, spleen, and blood—of rats, mice, pigs, dogs, and humans, as well as erythrocytes from twelve different animals. The rat tissue model uses respiration-rate data per tissue taken from,^49^ where brain, heart, kidney, and liver account for, respectively, 3%, 3%, 7%, and 20% of whole-body respiration. Details of the data compilation are provided in compiled measurements of ATP content per unit biomass across the kingdoms of life and data compilation from the literature for the theoretical model, where all relevant references are listed. Densities are drawn using kernel density estimation from compiled literature data and model predictions with sensitivity analysis. Outliers are not reported because, in this study, apparent outliers in the experimental data lack statistical meaning: each data point originates from a different study, organism, and set of physiological and environmental conditions, rather than representing variation within a single, comparable population. This figure simply shows the median turnover time. For the full range of simulated turnover times, see Table 1.

While biological mechanisms regulating ATP turnover time have been proposed, including those maintaining the homeostatic balance among ATP, ADP, and AMP concentrations,^34^^,^^35^ no quantitative physical model has been established in the literature to determine ATP turnover time. We address this problem using two complementary approaches. First, we compile data on ATP content across a wide range of organisms across the kingdoms of life (including ATP content in various animal tissues; compiled measurements of ATP content per unit biomass across the kingdoms of life) and divide it by our theoretically modeled ATP production rate, *J*_ATP_ (Equation 1), to estimate ATP turnover time. We find that the median ATP turnover time is less than 1 s in microalgae, higher plants, and fast-growing bacteria, but it can vary by up to two orders of magnitude in heterotrophic protists and animals (Figure 1; Table 1, discussion section). To shed light on the biophysical basis of these estimated values, we use cell size data for unicellular organisms and the range of cell sizes in animals,^36^ along with literature reports on mitochondrial positioning^37^^,^^38^ and population,^39^^,^^40^^,^^41^ and then examine how ATP diffusion time shapes overall ATP turnover time. To explore this further, we categorize organisms into three groups: (1) bacteria and cyanobacteria, (2) protists (both heterotrophic and photoautotrophic), and (3) multicellular organisms, including animals and plants.

**Table 1:** Summary of C:ATP ratios and ATP turnover times across organismal groups

Organism groups	Median C:ATP	***τ***_**min**_(**s**)	***τ***_median_ ± MAD(**s**)	***τ***_**max**_(**s**)
Cyanobacteria	404	0.001	0.52 ± 0.42	10.7
Green Algae	312	0.13	0.83 ± 0.32	3.53
Diatoms	302	0.02	0.78 ± 0.34	6.60
Dinoflagellates	311	0.05	0.63 ± 0.24	2.09
Plants	3743	0.03	0.21 ± 0.09	1.00
Leaves	3051	0.01	0.14 ± 0.08	1.28
Bacteria	256	0.01	0.54 ± 0.43	31.9
Ciliates	72	17.0	45.1 ± 10.5	110
Copepods	116	10.6	27.4 ± 5.20	64.6
Insects	44	0.66	1.96 ± 0.42	5.40
Fishes	106	46.9	103 ± 18.6	232
Nematodes	118	52.5	90.4 ± 14.2	188
Amphipods	100	12.2	28.4 ± 4.71	58.9
Amphibians	46	33.7	138 ± 41.1	494
Birds	43	5.43	11.6 ± 1.93	23.7
Mammals	121	2.40	5.95 ± 1.51	20.6
Rat brain	167	1.48	2.51 ± 0.38	4.49
Rat heart	53	2.51	4.06 ± 0.61	7.14
Rat kidney	151	0.46	0.74 ± 0.11	1.28
Rat liver	134	0.78	1.25 ± 0.19	2.21

Turnover times (median, minimum, and maximum) are calculated as the ratio of the experimental ATP pool to the theoretical ATP production rate, with theoretical estimates derived from our model using 10,000 random parameter samples. MAD denotes the median absolute deviation and is preferred over standard deviation because it is robust to outliers and skewed distributions, providing a more reliable measure of variability for non-normal data.

Heterotrophic bacteria are typically ∼1 μm in diameter, whereas photoautotrophic cyanobacteria reach ∼4 μm. Given these small cellular dimensions, the high copy number of ATP synthases in fast-growing aerobic bacteria, and the fact that ATP synthase complexes diffuse dynamically across the cytoplasmic membrane,^42^ we do not expect any substantial diffusion barrier for ATP transport from its sites of production to sites of consumption. ATP diffusion times calculated from both the free-diffusion and target-search formulations (see steady-state ATP turnover time) fall well below 1 s. This is further supported by our complementary approach, in which Equation 1 is used to estimate turnover time by dividing the experimental ATP pool by the theoretical *J*_ATP_, yielding median turnover times below 1 s for both bacteria and cyanobacteria (see Figure 1). This is consistent with experimental data showing ATP turnover times of less than 1 s during exponential growth and nearly 2 s during basal growth in fast-growing bacteria.^9^^,^^30^

Heterotrophic protists are, on average, substantially larger than bacteria and cyanobacteria, and most of their ATP is produced by mitochondria. Consequently, several factors become important determinants of ATP diffusion and thus ATP turnover time, including overall cell size, the number of mitochondria, their spatial distribution, the ATP consumption rate of different organelle types, and the intracellular packing density. A study of 13 heterotrophic protist species revealed that in cells larger than 10 μm, approximately 85% of mitochondria are located near the cell’s outer membrane,^37^ concluding that rapid access to oxygen is a key constraint shaping mitochondrial spatial distribution. Constructing an explicit model of ATP turnover time that incorporates all of these variables is beyond the scope of this paper, and free-diffusion and target-search formulations can provide only conceptual lower- and upper-bound estimates (see steady-state ATP turnover time). Thus, to obtain a more meaningful estimate of ATP turnover time in heterotrophic protists, we divide the empirical measurements of ATP content by our theoretical ATP production rates. As a representative group of heterotrophic protists, we examined ciliates, for which our results indicate turnover times ranging from 18 to 114 s, with a median of 45 s (see Figure 1; Table 1). For comparison, the literature reports an upper bound of 30 s for ATP turnover time in *Paramecium tetraurelia,* which falls within the range of our estimates.^31^

Photoautotrophic protists are, on average, similar in size to heterotrophic protists, and their respiration rates are also comparable (see, for example, the respiration rates of ciliates and dinoflagellates in Table S6). However, two key differences distinguish these groups. First, chloroplasts can occupy up to 45% of the total cell volume in photoautotrophs^38^^,^^39^ and produce their own ATP to satisfy local energy demands, reducing the volume of the cell that relies on mitochondrially produced ATP. Consequently, the effective respiration-to-volume ratio increases, which in turn lowers the ATP turnover time. The second major difference is that chloroplasts continuously generate oxygen via photosynthesis, creating an oxygen-rich intracellular environment. This enables mitochondria to position themselves close to chloroplasts, ensuring both immediate access to oxygen and proximity to other organelles, thereby minimizing ATP diffusion distances. Such mitochondrial positioning is consistent with three-dimensional reconstructions of intracellular organelle organization in microalgae.^38^ Our comparison of the theoretical ATP content model with experimental ATP measurements supports these conclusions, indicating that median ATP turnover times in microalgae are typically less than 1 s (see Figure 1; Table 1). ATP turnover time in higher plants is generally comparable to that observed in microalgae. As supported by our comparison between the ATP model and empirical ATP measurements, median ATP turnover times in higher plants can still remain below 1 s (see Figure 1; Table 1). This is consistent with experiments reporting ATP turnover times below 1 s in *Arabidopsis*.^30^^,^^32^

ATP turnover time in animals should be comparable to that in heterotrophic protists. Therefore, if one has data on the range and distribution of cell sizes that compose the body of an animal, it is possible to estimate the average ATP diffusion time. For instance, the size distribution and cell counts across all human tissues have recently been reported.^36^ In adult males, individual cell mass ranges from 9.00 × 10^−12^ g to 7.23 × 10^−4^ g, with a weighted mean (excluding non-nucleated cells) of 5.83 × 10^−9^ g,^36^ corresponding roughly to a size range of 2.6 μm–1114 μm, with a mean cell size of 22.3 μm. This average size is notably smaller than that of ciliates, typically reported around 100 μm.^43^ This suggests that the average ATP turnover time in humans could potentially be shorter than in ciliates. However, another key factor must also be considered. Whole-body maximum respiration rates in animals vary widely from relatively low rates in fishes to significantly higher rates in terrestrial insects (see Figure S3; Table S6). Although we earlier assumed that, under steady-state conditions, ATP production and consumption rates are equal, implying that respiration rate does not directly affect ATP turnover time, there is an important indirect effect. Consider two animals of the same size but with different respiration rates: The one with the higher rate will likely have a greater total mitochondrial volume or a higher mitochondrial population. Insects, for example, can have mitochondrial volumes reaching up to 43% of total cell volume,^41^^,^^44^ enabling mitochondria to be strategically positioned, with some near capillaries for optimal oxygen access and others near organelles for rapid ATP delivery. This mitochondrial arrangement partly explains why, according to our ATP model and its comparison with empirical ATP data, insects exhibit a median ATP turnover time of approximately 2 s, while fishes, which have lower respiration rates, show median turnover times around 103 s. Additionally, in animals, oxygen diffusion from the circulatory system (e.g., blood vessels) to individual cells presents another constraint on ATP production rate. For estimated turnover times across broad animal groups, see Table 1. For further discussion, see steady-state ATP turnover time.

### Carbon content

Carbon content $\left(C,\frac{\text{gram}}{\text{organism}}\right)$ ranges from as low as 5% of dry mass in ctenophores^45^ to as high as 65% in the leaves and stems of plants,^46^ but generally falls between 40% and 50% of an organism’s dry mass. Given that carbon turnover time is much longer than ATP turnover time, meaning carbon content remains relatively stable over timescales during which ATP levels fluctuate across different physiological states, we estimate carbon content using established equations that relate organism’s dry mass $\left(DM,\frac{\text{gram}}{\text{organism}}\right)$ to carbon content (see data compilation from the literature for the theoretical model). Structural materials $\left(SM,\frac{\text{gram}}{\text{organism}}\right)$, such as bacterial cell walls or vertebrate skeletons, can be either metabolically active (during synthesis, remodeling, degradation, motility, and so forth) or inactive depending on context. Since our goal is to estimate metabolically active organismal carbon content, we partially exclude *SM* from the organism’s *DM* before calculating carbon content. To do this, we use experimentally measured values of the maximum *SM* mass relative to *DM* obtained from the literature (see Table S5; Figure S5). We then consider a range of *SM* values, from zero, where the entire organismal mass is metabolically active, to the measured maximum, where *SM* is entirely inactive and should be excluded from *DM*. The equation used to calculate carbon content, *C*, is:

(Equation 4) $$C=a{\left(DM-SM\right)}^{b}$$

where *a* and *b* represent the scaling coefficient and scaling exponent, respectively.

## Discussion

Experimental measurements closely align with our model predictions across a wide range of photoautotrophs (cyanobacteria, green algae, diatoms, dinoflagellates, and plants), heterotrophs (bacteria, ciliates, copepods, amphipods, nematodes, insects, fishes, amphibians, birds, and mammals), and tissues (rat brain, heart, kidney, and liver, as well as leaves), with good agreement in both the median C:ATP values for each group as well as the range of variation within groups (Figure 1).

Across major biological groups, prokaryotes have a median C:ATP of 256 but exhibit extremely wide variation—from as low as 28.4 in exponentially growing *Streptococcus bovis* to as high as values reported for starved SAR11, for which ATP content measurements from three different studies yield C:ATP ranges of 437.5–22,500, 1741–6215, and a surprising 81,448–1,800,000 (SAR11 is a slow-growing bacterium and one of the most abundant species in surface ocean waters^47^) (Figure 1 and compiled measurements of ATP content per unit biomass across the kingdoms of life). Eukaryotes show a broad range of median C:ATP values across groups and tissues, from as low as 43 in birds to as high as 3,743 in plants. Within each group, the variation is even more substantial: C:ATP ranges from 1.2 in the brain of *Helicoverpa armigera* during the non-diapausing pupal stage to 47,801 in a deep-sea sponge. The major differences between prokaryotes and eukaryotes arise from: (1) the exceptionally strong size dependence of respiration rates in fast-growing prokaryotes with a scaling exponent near 2 for heterotrophic bacteria, making respiration far more sensitive to cell-size variation than in eukaryotes (this size dependence is further reflected in the normalized ATP content data, see compiled measurements of ATP content per unit biomass across the kingdoms of life); and (2) the presence of mitochondria and chloroplasts in eukaryotes, combined with their broader range of cell sizes, which together shape the diverse ATP turnover times observed across eukaryotic groups.

The C:ATP ratio is, on average, higher in eukaryotic photoautotrophs than in eukaryotic heterotrophs, largely due to the close *spatial* coupling of chloroplasts, mitochondria, and other organelles, which facilitates rapid oxygen access and short ATP turnover times. In prokaryotes, the C:ATP ratio in photoautotrophic cyanobacteria is higher than in heterotrophic bacteria primarily because fast-growing heterotrophic bacteria have substantially higher respiration rates than cyanobacteria at comparable cell sizes. Our dataset also indicates that nitrogen-fixing cyanobacteria have, on average, a lower C:ATP ratio than non-nitrogen-fixing strains, consistent with their elevated respiration rates required to meet the ATP demands of nitrogen fixation. Among photoautotrophs, plants exhibit substantially higher C:ATP ratios than microalgae (Figure 1). This arises from their much larger cell-size range,^48^ which yields diverse ATP turnover times across cells, different respiratory demands, and a high proportion of structural biomass—cell walls alone can constitute more than 90% of plant dry mass. As shown in Figure 1, our model predicts that plants have, on average, a median ATP turnover time of about one-third that of microalgae.

Diatoms and dinoflagellates have generally similar C:ATP ratios, although diatoms tend to have slightly lower values. Several factors contribute to this difference. First, diatoms have a slightly larger median size than dinoflagellates. They also exhibit lower respiration rates (by a factor of 3–4 for median-sized species), higher photosynthesis rates (approximately 2-fold on average), and greater SM content (also about 2-fold higher on average). Together, these factors suggest that diatoms have a slightly lower C:ATP ratio than dinoflagellates.

Our theoretical C:ATP model for animals represents whole-body C:ATP because whole-body respiration rates are widely available across animal groups, whereas tissue-specific respiration data are limited to a few cases (primarily in mammals). In contrast, most experimental C:ATP values reported for animals are tissue-specific rather than whole-body measurements, and they vary with the metabolic activity of each tissue, which differs substantially among species. Converting these tissue-specific experimental values to whole-body C:ATP would require detailed information on cell counts and cell size distributions across all tissues—data that are largely unavailable in the literature, with the notable exception of recent estimates for humans.^36^ Consequently, in Figure 1, we group experimental tissue-specific C:ATP values across different tissues within each animal category and treat them as representative estimates of whole-body experimental C:ATP for that group. Among the few cases where both tissue-specific ATP pools and respiration rates are available (such as in rats) we report both the theoretical and experimental tissue-specific C:ATP values in Figure 1 and Table 1. Details of these calculations are provided in tissue-specific respiration rates in animals. For example, in rats the ratio of “% body oxygen use” to “% body mass” ranges from 0.71 for skeletal muscle (30% O_2_ use/42% mass) to 7.78 for the kidney (7% O_2_ use/0.9% mass), spanning more than an order of magnitude.^49^ In humans, the pattern shifts: Skeletal muscle has the lowest ratio (0.48), whereas the heart has the highest (27.5), a >50-fold difference.^49^ Beyond organ-level respiration rates, ATP turnover time also varies substantially across tissues, as shown in Figure 1 and discussed in the steady-state ATP turnover time. ATP turnover time depends on multiple factors, including the type of circulatory system, the distance between blood vessels and target cells, the distribution of cell sizes within the tissue, and the mitochondrial population and spatial organization.

Holm-Hansen, the pioneer of the “universal C:ATP” concept, reported from 30 algal cultures that the ratio was independent of cell size.^50^ In contrast, using our compiled dataset spanning a broad range of organisms (see compiled measurements of ATP content per unit biomass across the kingdoms of life), we observed statistically significant size-related patterns in C:ATP for bacteria, microalgae, ciliates, insects, amphibians, birds, and mammals. Importantly, these correlations differ in sign: in some groups log_10_(C:ATP) decreases with log_10_(size), while in others it increases. Our current theoretical framework is a size-dependent model in all respects except for ATP turnover time, which we treat as a species-specific constant in our analysis. Understanding the origin of size-dependent C:ATP patterns requires a size-dependent model of ATP turnover time, which in turn would need data on cell sizes, mitochondrial abundance and spatial organization, intracellular packing density, and ATP consumption rates of different organelles. Developing such a model is beyond the scope of this paper. Given this limitation, we propose that the observed patterns of log_10_(C:ATP) versus log_10_(organism or tissue size) reflect, at least in part, the balance between two scaling relationships: (1) respiration rate versus size and (2) carbon content versus size (see on the size-dependence of C:ATP). For example, Table S6 shows that in bacteria the scaling exponent for respiration rate versus dry mass is 1.96, whereas the exponent for carbon content versus dry mass is 1.22. Because respiration increases more steeply with size than carbon content, log_10_(C:ATP) should decrease with increasing log_10_(bacterial cell size)—precisely matching the trend observed in our compiled dataset (for comparison, see Figure S2 versus Figure S6). However, this agreement cannot be generalized to other groups because the size dependence of ATP turnover time is not explicitly represented in the current model.

Experimental data show substantial variation in C:ATP across multiple groups, reflected in the skewed distributions (Figure 1). Here we highlight a few representative examples; full details of the physiological and environmental conditions under which ATP was measured are provided in compiled measurements of ATP content per unit biomass across the kingdoms of life. In insects, the left-tailed distribution is driven by the high ATP content of the cotton bollworm brain during the pupal stage, illustrating how ATP levels change markedly across developmental stages. In amphipods, the right-tailed distribution is caused by the low ATP content of *Gammarus lacustris* exposed to gradual warming in saltwater at 9 °C. Among mammals, the highest ATP content is reported in epididymal adipose tissue of rats, whereas the lowest is found in horse erythrocytes.

The C:ATP ratio may vary further under non-equilibrium conditions, which are not considered in the present work. Studies show that ATP content fluctuates with growth phase, carbon source, and cell cycle dynamics.^51^^,^^52^ For instance, *E. coli* and *P. putida* exhibit transient ATP accumulation during the shift from exponential to basal growth, due to a faster drop in ATP consumption than production especially pronounced with acetate or oleate as carbon sources.^51^ Single-cell studies reveal that ATP levels in *E. coli* can fluctuate 1.5- to 2-fold during exponential growth, with more stable ATP in fast-growing cells and greater fluctuations in slower-growing ones.^52^ These changes are tied to the cell cycle, mitochondrial dynamics, and cyclin-dependent kinase activity.^53^^,^^54^

Patel et al.^55^ proposed that ATP acts as a hydrotrope (in addition to its role as the universal energy currency), requiring millimolar concentrations to keep proteins soluble despite enzymes needing only micromolar levels. Bochdansky et al.^6^ noted that this explains the universally conserved cytoplasmic ATP concentration, making it a reliable proxy for microbial cytoplasmic volume and biomass in ocean studies. Aside from the proposition that ATP may act as a hydrotrope, the arguments above face several criticisms. First, as we document in this paper, the normalized ATP concentration is not universally millimolar but instead varies widely across the tree of life (see the normalized ATP content compiled in compiled measurements of ATP content per unit biomass across the kingdoms of life showing variation by five orders of magnitude). Second, the micromolar ATP requirements of enzymes cannot account for the total rate of ATP consumption, because a major fraction of cellular energy is dissipated as heat due to the inefficiency of intracellular machineries.^56^ The actual rate of ATP consumption at the level of cellular traits and organelles remains an active field of study with many unknowns.^2^ Third, Patel et al.^55^ provide a new role for ATP but do not address why cells maintain substantial intracellular ATP pools. As we argue here, the accumulation of ATP in cells is best explained by ATP turnover time, which varies extensively across organisms and depends on at least cell size, mitochondrial abundance and spatial distribution, intracellular packing density, and the ATP consumption rate of each organelle type.

### Conclusion

Quantifying biomass is fundamental to understanding bioenergy resources, carbon cycling, ecosystem productivity, resource management, and climate change.^57^^,^^58^ The carbon-to-ATP (C:ATP) ratio has long been used as a proxy for marine microbial biomass based on the assumption of a universal value of ∼250. However, despite its widespread use, the origin and validity of this “canonical” number have remained unclear. By compiling ∼400 measurements across the kingdoms of life, we show that C:ATP is not constant but varies by five orders of magnitude across species, physiological states, and environmental conditions, indicating that treating it as a fixed constant can lead to substantial errors in biomass estimation.

To resolve this discrepancy, we developed a simple mechanistic framework that both recovers the value of ∼250 under specific conditions and explains its broad variability. In our model, C:ATP is determined by metabolic rate, ATP turnover time, and functional carbon content, revealing that the canonical value is not universal but emerges from particular physiological regimes.

Despite large variability, the median C:ATP for bacteria and unicellular marine eukaryotes remains within a factor of two of ∼250, somewhat consistent with historical observations. Systematic deviations occur across the tree of life, with lower values in multicellular animals and higher values in plants. In photosynthetic eukaryotes, reduced ATP transport distances (due to the proximity of mitochondria and chloroplasts to one another) contribute to elevated C:ATP relative to heterotrophs.

A key contribution of this work is establishing the biophysical meaning of ATP turnover time. We hypothesize that it is governed by cell size, mitochondrial abundance and spatial organization, intracellular packing density, and ATP consumption rates across organelles, thereby linking subcellular structure to organismal energetics. A first-principles model of ATP turnover time (grounded in fundamental physical laws) remains an open challenge. Separately, future work should quantify how environmental factors, including temperature and nutrient limitation, shape C:ATP variability.

Overall, this work demonstrates that the C:ATP ratio should be treated as a context-dependent quantity, determined by species composition, physiology, and environment. This work shifts C:ATP from an empirical constant to a mechanistically grounded and predictive framework for estimating biomass across biological systems.

### Limitations of the study

Our mechanistic model provides an idealized first-step framework and does not incorporate all influencing variables. Although factors such as species-specific respiration rate, organism size, growth rate, carbon source, metabolic workload, and stress exposure are included explicitly or implicitly through Equations 1, 2, and 3, other factors such as developmental stage (e.g., age, cell division rate, reproductive state) and environmental drivers including temperature and nutrient limitation are not explicitly modeled and remain important directions for future work.

## Resource availability

### Lead contact

Peyman Fahimi; email address: fahimi@dal.ca.

### Materials availability

Figures S1–S7 and Tables S1–S7 cited in the main text and STAR Methods are available in the Supplementary Information PDF.

### Data and code availability

• The supplementary data (Data S1) are available on Mendeley Data. Link: https://data.mendeley.com/datasets/j4bwxwy6gz/1.
• All Python source code used to generate the figures and tables reported in this study is available from the lead contact upon request.
• Any additional information required to reanalyze the data reported in this paper is available from the lead contact upon request.

## Acknowledgments

This work was supported by the Simons Collaboration on Computational Biogeochemical Modeling of Marine Ecosystems/CBIOMES (grant nos. 549937, Z.V.F. and 549935, A.J.I.). The authors thank Dr. Manon Laget, Dr. Dany Croteau, Dr. Niall McGinty, and Dr. Ruby Hu (all from Dalhousie University) for helpful discussions, and the editor and three anonymous reviewers for their constructive comments.

## Author contributions

P.F. developed the theoretical framework, performed all analyses, assembled and curated the data, and wrote the manuscript. M.M.A. contributed to the statistical analysis, discussions, and edited the manuscript. A.J.I. and Z.V.F. proposed the initial research direction, contributed through discussions and critical feedback, secured funding, and edited the manuscript. All authors approved the final version.

## Declaration of interests

The authors declare no competing interests.

## Declaration of generative AI and AI-assisted technologies in the writing process

During the preparation of this work, ChatGPT-5.5 was used to assist in the creation of the graphical abstract.

## STAR★Methods

### Key resources table

**Table:** 

REAGENT or RESOURCE	SOURCE	IDENTIFIER
**Deposited data**		

Data S1: C:ATP dataset	This study	Mendeley Data:https://data.mendeley.com/datasets/j4bwxwy6gz/1

**Software and algorithms**		

Jupyter Notebook	Project Jupyter	Version 7.4.4
Python	Python Software Foundation	Version 3.11.5
Python source code	This study	Available from the lead contact upon request

### Experimental model and study participant details

Omitted as our study does not involve biological models.

### Method details

#### Compiled measurements of ATP content per unit biomass across the kingdoms of life

We compiled 397 ATP-content measurements from 82 papers published between 1964 and 2024, spanning a wide diversity of taxa. The dataset encompasses bacteria (9 classes, *n* = 108 data points), microalgae (13 classes, *n* = 113), heterotrophic protists (1 class, *n* = 16), arthropods (3 classes, *n* = 24), Mollusca and nematodes (3 classes, *n* = 9), insects (*n* = 17), amphibians (*n* = 8), fishes (2 classes, *n* = 6), birds (*n* = 7), mammals (*n* = 56), sponges (*n* = 1), and plants (*n* = 32). Across animal and plant tissues, the data include muscle (*n* = 34), brain (*n* = 11), heart (*n* = 17), kidney (*n* = 2), liver (*n* = 5), fat body (*n* = 5), blood and red blood cells (*n* = 20), spleen (*n* = 1), sperm (*n* = 3), leaves (*n* = 22), and roots, shoots, and other plant cell lines (*n* = 10). All data are provided in the Supplemental Data S1.

Literature screening and data curation were conducted through an iterative search strategy during 2024–2025 using Google Scholar. The compilation was initially built from ATP datasets and references reported in review articles and primary studies on cellular ATP content and biomass estimation. Additional studies were then identified through forward and backward citation tracking, as well as targeted organism-specific searches (e.g., bacteria, protists, animal tissues, and plants) to broaden taxonomic and physiological coverage. Search terms included combinations of keywords such as “carbon/ATP ratio”, “C:ATP ratio”, “microbial biomass”, “ATP content”, “ATP concentration”, “ATP pool”, “particulate ATP”, “adenosine triphosphate”, “adenine nucleotide pool”, and organism- or taxon-specific terms. The compiled dataset is not uniformly distributed across publication years, with a substantial fraction of studies originating from the 1970s. This pattern reflects periods of intensive experimental work on ATP quantification during the widespread adoption of luciferin–luciferase assays and related extraction protocols.

Studies were included if they (i) reported experimentally measured ATP values obtained using established biochemical or analytical methods, (ii) provided sufficient contextual information to relate ATP content to biomass, physiological state, or environmental conditions, and (iii) represented distinct taxa, tissues, physiological states, or environmental conditions to capture broad biological variability. Studies lacking quantitative ATP measurements, sufficient methodological/contextual information, or presenting duplicated datasets were excluded where identifiable.

For each study, the file includes: (1) reference; (2) ATP assay method; (3) potential methodological caveats; (4) physiological factors affecting ATP content; (5) class; (6) group; (7) common name/species; (8) tissue group; (9) physiological state for which ATP content is reported; (10) original ATP units; (11) ATP content in original units; (12) ATP in grams per gram of dry mass; (13) tissue or cell dry mass (g); (14) carbon content (g); (15) ATP per cell or tissue (g); (16) C:ATP ratio; and (17) method used to estimate tissue or cell dry mass. About 55% of the included studies do not systematically report methodological caveats.

For each species or tissue, we report the minimum and maximum ATP content in the original units, then convert these values to a common unit (g ATP per g dry mass) to enable cross-study comparison. This approach captures the full extent of variability, aligned with the aim of this study, rather than solely relying on representative mean or median values. Note that these values correspond to observed measurements and do not reflect the experimental errors reported in the original studies. As illustrated in Figure S1, normalized ATP content spans 2.78×10^-7^ to 4.46×10^-1^ g ATP g^-1^ dry mass, representing over five orders of magnitude variation.

We do not standardize ATP content to a common temperature for two reasons. First, one of the central objectives of this study is to demonstrate that ATP per unit biomass varies with environmental factors despite its traditional treatment as constant. Second, ATP content does not obey a universal temperature-scaling relationship comparable to that of respiration rate. As reflected in Equations 1, 2, and 3, multiple parameters govern the temperature dependence of the ATP pool, and their individual responses to temperature must be explicitly modeled in future work. Consistent with this, experimental studies show that ATP and carbon content can increase, decrease, or remain unchanged with temperature, depending on organism, growth phase, stress type, acclimation state, and environmental conditions.^59^^,^^60^^,^^61^^,^^62^^,^^63^^,^^64^

Because most studies did not report total organism or organ mass, we estimated masses using several approaches described in Supplementary Data S1 (see the column “How the tissue or cell dry weight is estimated?”). For example, in mammals, organ masses were estimated using published scaling relationships that relate whole-organism mass to organ-specific mass. These estimates allowed us to evaluate how the C:ATP ratio scales with organism or tissue dry mass. As shown in Figure S2 and Table S1, the C:ATP ratio exhibits statistically significant size-dependent patterns in several groups, based on the *p*-values of the regression slopes of log_10_(C:ATP) versus log_10_(dry mass). In bacteria, microalgae, birds, and mammals, the correlation is negative, whereas in insects, ciliates, and amphibians, it is positive. Across tissues, statistically significant patterns are observed in erythrocytes and leaves (negative correlation) and in brain tissue (positive correlation).

#### Data compilation from the literature for the theoretical model

To estimate C:ATP ratios, we assembled dry-mass ranges (Table S2) and collated equations predicting respiration rate, carbon content, and structural material composition based on organism size (Tables S3–S5), then we standardized the units for all variables (Table S6) across a diverse set of organisms including bacteria, microalgae, heterotrophic protists, multicellular zooplankton, freshwater invertebrates, terrestrial insects, nematodes, fishes, amphibians, birds, mammals, and vascular plants. The procedure used to perform the standardization is described in detail in standardization of respiration rates and carbon content across units and temperature. To study the distribution around the median C:ATP ratio, we performed a sensitivity analysis for all parameters with a range of values. We randomly selected 10,000 samples using a normal distribution centered around the mean or median value, bounded by the minimum and maximum observed values. If the mean or median was unavailable, we used a uniform distribution instead. We compared the results of our C:ATP model with experimental measurements (∼400 data points from 82 papers published between 1964 and 2024; Supplementary Data S1).

##### Range of dry mass in studied organisms

Table S2 summarizes the dry mass range of all organisms included in this study. Reported dry mass values were extracted directly from the original references whenever available. For organisms lacking direct measurements, dry mass was estimated from body mass using standard assumptions regarding body composition and density. These values were used in all subsequent analyses.

##### Aerobic respiration rate

We have compiled empirical scaling equations from various sources that relate respiration rates in heterotrophs and dark respiration rates in photoautotrophs to organism size (Table S3). These equations apply to organisms in an actively growing state for protists and prokaryotes, food-saturated growth rates for multicellular zooplankton, maximum growth rates for freshwater invertebrates, maximum metabolic rates for vertebrates and terrestrial insects, a naturally growing state for field tree saplings, and growth in nitrogen-enriched soil for greenhouse tree seedlings. We retain the original units provided by each reference in Table S3 but standardize all units to a unified format in Table S6. Additionally, we convert all the original temperatures to 25°C using a Q_10_ value of 2.5.^65^ For endothermic vertebrates, we fix the temperature at 38°C. Where data or equations relating organism volume to dry mass are unavailable, we use the relationship $DM\left[\text{pg}\right]≅0.57{\left(V\left[\mu m^{3}\right]\right)}^{0.92}$, as developed by Lynch.^1^ If equations are provided in terms of body mass, we assume that dry mass is 30% of the body mass.^13^ Figure S3 depicts the respiration rate in both heterotrophs and photoautotrophs as a function of dry mass, illustrating how respiration rate varies across species. The dry mass in the figure spans 20 orders of magnitude.

##### Carbon content across the kingdoms of life

Carbon turnover time is significantly higher than ATP turnover time, meaning carbon content remains stable over timescales in which ATP content fluctuates. This stability allows us to compute carbon content from dry mass using power-law functions. This approach is reliable due to the strong correlation observed, as evidenced by the high R^2^ values in the empirical equations (Table S4). An alternative method estimates carbon content using maximum growth rate scaling equations. While this method offers insights into carbon content increase per unit time, it typically produces lower R^2^ values, ranging from as low as 0.001 in larval fish to 0.733 in crustaceans, with other organisms falling between these extremes.^66^ Consequently, we have compiled data on the mass-scaling of carbon content per organism from various sources, with the summarized results presented in Table S4. The same equations, standardized to consistent units, are provided in Table S6. Figure S4 compares the carbon content versus dry mass across different organisms, showing those with a known power-law relationship (top panel) and those for which only the carbon-to-dry-mass ratio is available (bottom panel).

The literature commonly excludes structural materials (SM) including frustules, theca, lorica, coccoliths, siliceous scales, cell walls, tunics, chitin, shells, molts, and skeletons from dry mass prior to carbon estimation, on the assumption that these components are metabolically inert once formed. However, this assumption is not universally valid. Structural materials can remain metabolically relevant after formation through synthesis, remodeling, repair, degradation, turnover, and functional interactions with growth, division, protection, and motility. This is particularly clear for organic structural materials such as bacterial cell walls, chitinous structures, tunics, molts, and skeleton-associated matrices, but even inorganic structural materials such as frustules and coccoliths are biologically produced and regulated rather than purely passive materials.

To evaluate the impact of SMs on C:ATP ratios, we therefore treat the metabolically relevant contribution of SM as variable rather than fixed. Instead of assuming that SM is either fully excluded or fully included, we implement a bounded uncertainty approach in which the structural fraction of dry mass can vary continuously between zero and an upper limit derived from the literature. Specifically, the maximum reported contribution of SMs to organism dry mass, summarized in Figure S5 and Table S5, is used to define the upper bound.

In the simulations, the fraction of dry mass allocated to metabolically inactive SM is randomly sampled from a uniform distribution between 0 and this maximum value. This reflects the fact that, over an organism’s life cycle, the metabolic engagement of SM can vary from minimal to substantial depending on growth stage, remodeling, repair, degradation, and physiological state. The metabolically active dry mass entering the C:ATP calculation is then computed by subtracting this sampled inactive structural fraction from total dry mass. This adjusted dry mass is used in Equation 4 to estimate carbon content.

The exclusion of storage oils or lipid droplets, referred to as non-living carbon, from carbon estimations has been proposed.^67^ However, while lipid storage may sometimes be temporarily metabolically inactive, it remains highly dynamic and potentially active, contributing to numerous cellular processes. Recent research has highlighted their roles in metabolism, energy homeostasis, signaling, and buoyancy regulation, among others.^68^^,^^69^^,^^70^^,^^71^ In eukaryotes, lipid droplets establish active interactions with organelles such as the endoplasmic reticulum, mitochondria, Golgi apparatus, and vacuoles, which are essential for the normal cellular life cycle.^68^ The copy number and size of these droplets also respond dynamically to the type of food (carbon source) and changes in cell volume.^40^ In prokaryotes, lipids like poly(3-hydroxybutyrate), polyhydroxyalkanoates, triacylglycerols, and wax esters play active roles in responding to stress, imbalanced growth, and starvation conditions.^70^ Hence, we include lipid storage mass in our estimates of carbon-to-ATP ratios.

##### Standardization of respiration rates and carbon content across units and temperature

To enable direct comparison across datasets, respiration rates (Table S3) and carbon content (Table S4) were standardized to the same units and normalized to the reference temperature provided in Table S6; here we elaborate how this standardization was mediated.

##### Respiration rates

###### Diatoms and cyanobacteria

The data are taken from,^13^ where dark respiration rates (DRR) are reported in units of watts per kilogram of wet mass, and body mass (BM) is given in picograms at 25 °C. We first converted DRRs to watts per organism by multiplying the original unit by BM. A reduced major axis (RMA) regression was then applied to obtain the equation presented in Table S3. The standardized equations in Table S6 are expressed in mol O_2_ per organism per second at 25 °C, with organism mass reported as dry mass (DM). For this conversion, we assumed that 1 ml O_2_ = 20 J, the molar volume of O_2_ = 22,400 ml/mol at STP, and DM = 0.3 BM.

###### Dinoflagellates, green algae, and golden algae

The data are taken from,^72^ where DRRs are reported in pl O_2_ per organism per hour, and organismal carbon content is given in picograms. An RMA regression was applied to derive the equation presented in Table S3. Although DRR versus carbon content is a more direct metric for estimating C:ATP ratios, we converted carbon to DM in order to provide equations consistent with those of other organisms for comparative purposes. For this conversion, we used the relationship *C*[pg]≅1.59×(*DM*[pg])^0.96^ developed for phytoplankton in Ref.^73^ The resulting equations are reported in Table S3. Finally, DRRs and DM were standardized to the units used in Table S6, applying the conversion factors described in the previous paragraph.

###### Heterotrophic protists and bacteria

The equations are taken from,^18^^,^^74^ where RRs are reported in watts per organism at 20 °C, with BM expressed in grams. To standardize the units, we applied the following conversions: 1 ml O_2_ = 20 J, the molar volume of O_2_ = 22,400 ml/mol at STP, DM = 0.3 BM, and Q_10_ = 2.5.

###### Multicellular zooplankton

The data are taken from,^14^ where RRs are reported in microliters of O_2_ per organism per hour at 15 °C, and carbon content is given in micrograms. We applied an RMA regression to derive the relationship between RR and carbon content. For consistency with other organisms, we converted carbon content to DM using the zooplankton-specific equations provided in Ref.^45^ Finally, we standardized the units to those used in Table S6 and adjusted the temperature to 25 °C using Q_10_ = 2.5.

###### Freshwater *Daphnia* and *Bosmina*

The equations are taken from,^75^ where average RRs are reported for *Daphnia* species and for *Bosmina longirostris* in microliters of O_2_ per organism per day at 20 °C, expressed as a function of DM in micrograms. The units were standardized to those used in Table S6 using the conversion factors described in the previous paragraphs.

###### Vascular plants

The equations are taken from,^76^ where whole-plant RRs of various plant types are reported in nmol O_2_ per plant per second at 24 °C as a function of DM in grams. In Table S6, these equations are converted to mol O_2_ per second and standardized to 25 °C.

###### Leaves

The equation is taken from,^77^ where RR for lettuce (*Lactuca sativa L*.) is reported in micromoles O_2_ per plant per second at 25 °C as a function of DM in grams. For consistency, RR was converted to moles O_2_ per second and presented in Table S6.

###### Insects

The equations are taken from,^78^ where RRs during flight are reported in mm^3^ O_2_ per organism per hour at 22 °C as a function of BM in milligrams. To convert mm^3^ O_2_ to moles O_2_, as reported in Table S6, it was assumed that 1 mole O_2_ = 22.4 × 10^6^ mm^3^ at STP. All other units were standardized to those in Table S6 using the same assumptions described previously.

###### Nematodes

The data are taken from,^79^ considering only species from oxic sites with respiration measured under oxic incubation. RRs were reported in nmol O_2_ per organism per day at 10 °C as a function of BM in micrograms. BM was first converted to DM using the relation DM = 0.3 BM, and an RMA regression was then applied to derive the equation presented in Table S3. Finally, units were standardized to those reported in Table S6 using the conversion factors described earlier.

###### Vertebrates

The data are taken from,^80^ where maximal RRs are reported in ml O_2_ per organism per hour at various temperatures as a function of BM in grams. Temperatures were normalized by converting ectotherm values to 25 °C and endotherm values to 38 °C using Q_10_ = 2.5. An RMA regression was then applied to obtain the equations presented in Table S3. Finally, units were standardized to those in Table S6 using the conversion factors described earlier.

##### Tissue-specific respiration rates in animals

In the right panel of Figure 1 of the paper, we present an example of modeling tissue-specific C:ATP ratios for rat brain, heart, kidney, and liver. To estimate tissue-specific respiration rates, we first calculate the whole-body respiration rate using the mammalian scaling equation provided in Table S6. We then obtain organ-specific fractions of total oxygen consumption in rats from the literature.^49^ Tissue-specific respiration rates are calculated by multiplying the whole-body respiration rate by the corresponding organ-specific fraction (e.g., 3% for brain). The ranges of whole-body dry mass used to estimate whole-body respiration rates, as well as the ranges of organ-specific dry mass used to estimate tissue carbon content in rats, are provided in Supplementary Data S1.

##### Carbon contents

###### Diatoms, dinoflagellates, and green algae

The equations are taken from,^73^ where carbon content per organism (pg) is reported as a function of cell volume (μm^3^). Dry mass (pg) is also reported as a function of cell volume (μm^3^). Combining these two equations allowed us to establish the relationship between carbon content and dry mass, as presented in Table S4. Both variables were then converted to grams to match the units reported in Table S6.

###### Golden algae, cyanobacteria, ciliates, and heterotrophic dinoflagellates

The equations are taken from,^81^ where carbon content (pg) per organism is reported as a function of cell volume (μm^3^), as shown in Table S4. We first converted volume to dry mass (DM) using the equation $V\left[\mu m^{3}\right]={\left(\frac{DM\left[\text{pg}\right]}{0.57}\right)}^{\left(\frac{1}{0.92}\right)}$, developed in Ref.^1^ for unicellular organisms, with the resulting equations provided in Table S4. We then standardized the units to match those in Table S6, where both carbon content and DM are expressed in grams.

###### Heterotrophic prokaryotes

The equation is taken from,^82^ where carbon content in femtograms per organism is reported as a function of cell volume in μm^3^ (see Table S4). We first converted volume to DM using the relation $V\left[\mu m^{3}\right]={\left(\frac{DM\left[\text{pg}\right]}{0.57}\right)}^{\left(\frac{1}{0.92}\right)}$, as provided in Table S4. We then standardized both carbon content and DM to grams, consistent with the units reported in Table S6.

###### Multicellular zooplankton

The equations are taken from,^45^ where carbon content per organism (mg) is reported as a function of wet mass (mg). Separately, dry mass (mg) is reported as a function of wet mass (mg). By combining these two equations, we derived the relationship between carbon content and dry mass, as shown in Table S4. Both carbon content and dry mass were then standardized to grams, consistent with the units reported in Table S6.

#### Proton leak in mitochondria and bacteria

Not all oxygen molecules respired are necessarily converted into ATP.^83^ Unregulated basal proton leak (PL) through adenine nucleotide translocases (ANT),^83^ and water wires (WW),^22^ as well as regulated inducible PL through uncoupling proteins (UCPs) and ANTs,^22^^,^^83^ dissipates a significant amount of energy as heat without ATP production. Presence of UCPs have been reported in mammals, birds, reptiles, amphibians, fishes, insects, plants, fungi, and protozoa,^84^ and PL has also been observed in bacterial cells.^85^^,^^86^ PL increases nonlinearly with inner mitochondrial membrane potential^21^^,^^87^ and correlates positively with the standard metabolic rate of organisms.^87^

Due to the limited data on the relationship between mitochondrial PL and metabolic rate across all kingdoms of life, we are not using scaling equations to estimate PL at this time. This is primarily because the electrochemical potential of the inner mitochondrial membrane (IMM) is highly dynamic, fluctuating in time and space under both physiological and stress conditions,^88^ as well as during the fission and fusion of mitochondrial networks.^89^^,^^90^^,^^91^^,^^92^ For example, the potential of the IMM is not homogeneous; it is higher in the cristae relative to the inner boundary membrane^93^ and is elevated at the sites of ATP synthase molecules due to the intrinsic molecular electrostatic potential of this enzyme.^94^ Additionally, the membrane potential is dynamically regulated through various metabolic pathways and electrical feedback mechanisms,^95^ making it challenging to develop an equation that accurately represents the relationship between respiration rate and proton leak.

Although proton leak has been documented across diverse groups of organisms, quantitative estimates of its magnitude as a fraction of respiration are available for only a limited set of animal tissues, primarily hepatocytes in mammals, birds, reptiles, amphibians, and fishes, as well as rat muscle and the hepatopancreas of snails.^17^ Applying these tissue-specific values to whole-organism respiration in our model may therefore introduce substantial bias, as proton leak can vary markedly among tissues. Moreover, we found no quantitative data on proton leak expressed as a percentage of respiration for bacteria, protists, or most other microbial groups, with the exception of yeast cells. Taken together, these limitations motivate a conservative modeling approach: we adopt the full range of values reported in the literature (10–50% of respiration) and apply it uniformly to mitochondrial respiration across organisms as well as to bacterial respiration.^17^^,^^22^ More research is required to quantify differences in proton leak across the domains of life.

#### Stoichiometric ratios of ATP/O_2_ in oxidative phosphorylation

The maximum number of ATP molecules produced per oxygen atom consumed $\left(\frac{\text{ATP}}{O^{c}}\right)$ in oxidative phosphorylation and glycolysis pathways^10^^,^^17^ is determined by the equation:

(Equation 5) $$\frac{\text{ATP}}{O^{c}}=\frac{\frac{H^{+}}{{\left(\frac{H^{+}}{\text{ATP}}\right)}_{\text{ATP}\;\text{synthase}}}+{\text{ATP}}_{\text{aerobic}\;\text{glycolysis}+\text{TCA}\;\text{cycle}+\beta \;\text{oxidation}}-{\text{ATP}}_{\text{activation}}}{O^{c}}$$

Here, the ${\left(\frac{H^{+}}{\text{ATP}}\right)}_{\text{ATP}\;\text{synthase}}$ ratio represents the number of protons translocated through ATP synthase to produce one ATP molecule. All other parameters depend on the specific metabolic pathway. For instance, during the oxidation of palmitate: O^c^=46, total H^+^=392, ATP_activation_=2, and ATP_TCA cycle+β oxidation_=8.^10^^,^^17^ The ${\left(\frac{H^{+}}{\text{ATP}}\right)}_{\text{ATP}\;\text{synthase}}$ ratio depends on the number of c-subunits in the ATP synthase complex. For example, the ATP synthase in the mitochondria of *Saccharomyces cerevisiae*,^96^ *Yarrowia lipolytica*,^97^ and *Euglena gracilis*^98^ contains 10 c-subunits, while in human,^99^ bovine,^100^ and pig^101^ mitochondria, it contains 8 c-subunits.^102^^,^^103^ Due to the absence of data on the number of c-subunits in the mitochondrial ATP synthase of all the organisms studied in this paper, we assume that mitochondrial ATP synthase in unicellular organisms contain 10 c-subunits, while mitochondrial ATP synthase in multicellular organisms contain 8 c-subunits. In heterotrophic bacteria, the number of c-subunits ranges from 9 to 17.^102^^,^^103^

For each ATP produced in mitochondria, one inorganic phosphate (Pi) is transported into the mitochondrial matrix via phosphate carriers (PiC), accompanied by one proton.^104^ Consequently, we must add three additional protons to the total from each c-subunit.^17^ This results in ${\left(\frac{H^{+}}{\text{ATP}}\right)}_{\text{ATP}\;\text{synthase}}$ ratios ranging from approximately (8+3)/3 ∼ 3.66 and (10+3)/3 ∼ 4.33. In bacterial ATP synthase, the F_1_ portion of the enzyme is located in the cytoplasm,^105^ where inorganic phosphate is already present. Therefore, no phosphate carrier (PiC) is required. As a result, the proton-to-ATP ratio for ATP synthase ${\left(\frac{H^{+}}{\text{ATP}}\right)}_{\text{ATP}\;\text{synthase}}$ ranges from 9/3 = 3 to 17/3 ∼ 5.66, depending on the number of c-subunits in the ATP synthase. Assuming that the metabolic pathways involved in oxidative phosphorylation and glycolysis are largely conserved across all kingdoms of life, with differences primarily in the number of c-subunits in ATP synthase, the Table S7 presents the minimum and maximum values of $\frac{\text{ATP}}{O^{c}}$ for c-subunits ranging from 8 to 17. To simplify future calculations, we double the values to obtain the ATP per molecule of oxygen $\left(\frac{\text{ATP}}{O_{2}^{c}}\right)$ in Table S7.

#### Photosynthesis rate, proton leak, and $\text{ATP/}O_{2}^{p}$ ratio in chloroplast

In photoautotrophic organisms, the ATP synthase in chloroplasts contains 14 c-subunits, as reported for *Spinacia oleracea*,^106^ *Pisum sativum*,^107^ and *Triticum aestivum*,^108^ whereas in *Synechococcus* cyanobacteria, it ranges from 13 to 15 c-subunits.^102^^,^^103^ Although no crystallographic or microscopic studies have reported an ATP synthase in chloroplasts with fewer than 14 c-subunits, it has been suggested that the chloroplast ATP synthase in *C. reinhardtii* may have 13 c-subunits instead of the usual 14. This suggestion is based on observed differences in the migration pattern of proteins in SDS-PAGE gels.^109^

In chloroplasts, during non-cyclic photophosphorylation or linear electron flow (LEF) under standard conditions, twelve protons are pumped into the thylakoid lumen for each oxygen molecule produced during photosynthesis,^110^ yielding an $\frac{\text{ATP}}{O_{2}^{p}}$ ratio of approximately 12 ÷ ((14 or 13) ÷ 3) ∼ 2.57 to 2.77. However, as discussed earlier and previously reported for mitochondria,^10^^,^^17^ each inorganic phosphate (Pi) used in ATP synthesis is accompanied by the transport of one proton from the cytoplasm into the chloroplast via the phosphate carrier (PiC), a factor often overlooked in chloroplast studies. Accounting for this, the adjusted $\frac{\text{ATP}}{O_{2}^{p}}$ ratio ranges approximately from 2.12 to 2.25. Under standard conditions, chloroplasts require at least three ATP molecules per two NADPH molecules to sustain carbon fixation alone.^16^ The additional ATP necessary for other processes is generated through cyclic photophosphorylation or cyclic electron flow (CEF), which does not involve oxygen and supports the higher ATP/NADPH ratio needed for the Calvin cycle,^111^ CO_2_-concentrating mechanisms,^112^^,^^113^^,^^114^ and other intra-chloroplast processes. Determining the proportion of ATP produced via CEF versus LEF depends on the rates of both pathways. Literature reports that the CEF-to-LEF ratio can range from 3%^111^ to 14%^110^ and up to 60%.^27^ However, it has been reported that the rate of electron transfer per second per PSI complex in CEF can reach up to 210 e^-^ s^-1^ PSI^-1^, suggesting that CEF can potentially operate as fast as LEF.^115^^,^^116^^,^^117^ Additionally, the pseudo-cyclic pathway, where molecular oxygen acts as the terminal electron acceptor in the electron transport chain, can generate ATP at rates as high as 20% of LEF.^27^ Thus, it is reasonable to assume that $\frac{\text{ATP}}{O_{2}^{p}}$ ranges from 2.12 + 2.12 + 0.2(2.12) = 4.66 to 2.25 + 2.25 + 0.2(2.25) = 4.95, accounting for the combined contributions of all LEF, CEF, and pseudo-CEF pathways.

Proton leakage across the thylakoid membrane of chloroplasts has been observed under specific conditions, such as low inorganic phosphate levels in isolated spinach and lettuce thylakoids.^118^^,^^119^ However, the extent of this leakage remains poorly studied, and the available literature reports inconsistent values. For example, in *Spinacia oleracea*, uncoupling of protons via the thiol reagent dithiothreitol or light has been reported to dissipate 20–30% of ATP yield.^120^ In contrast, a study on *Arabidopsis* leaves found the ion leakage to be negligible.^121^ Additionally, in young spinach leaves, ion leakage (though unspecified as to the type of ions) increased under dark conditions when the electrochemical proton gradient exceeded 190 mV.^122^ Therefore, our model assumes that proton leak dissipates between 0% and 30% of the ATP production rate in the chloroplast.

To estimate the total rate of ATP production in photoautotrophic organisms, it is necessary to account for all ATP-producing pathways, including mitochondrial pathways, glycolysis, and chloroplast-mediated pathways. A key missing element is the relationship between respiration and photosynthesis rates in microalgae and higher plants. The ratio of dark respiration rate to light-saturated photosynthesis rate $\left(\frac{DRR}{\mathrm{PR}}\right)$, defined as $=\frac{O_{2}^{c}}{O_{2}^{p}}$, varies across species. For actively growing cells, this ratio ranges from 0.08 to 0.6 in Dinophyceae (mean ∼ 0.35±0.17), 0.013 to 0.50 in Bacillariophyceae (mean ∼ 0.16±0.06), 0.043 to 0.63 in Chlorophyceae (mean ∼ 0.13±0.07), 0.03 to 0.64 in Prymnesiophyceae (mean ∼ 0.16±0.04), and 0.004 to 0.17 in Cyanophyceae (mean ∼ 0.10±0.07).^15^^,^^28^^,^^29^ It is observed that this ratio is independent of cell size^15^ and light intensity.^23^^,^^24^ For vascular plants, we use ratios associated with Chlorophyceae.

#### Steady-state ATP turnover time

As discussed in the main text, the steady-state ATP turnover time depends on the diffusion time of ATP from its site of production to its site of consumption. Here we explore quantitatively the factors known to affect ATP delivery time.

##### ATP diffusion coefficient

ATP molecules are produced in the mitochondrial matrix, where the viscosity is twice that of pure water, and their diffusion is significantly hindered by the presence of cristae membranes that vary across different respiratory states.^123^ Subsequently, ATP must traverse the highly selective mitochondrial membrane, where the ATP-ADP exchange rate depends on the membrane's electrochemical potential.^124^ At steady state, in liver mitochondria depolarized to different membrane voltages, the ATP exchange rate ranges from near zero at -100 mV to 0.5 mM ATP per minute per milligram of protein at -145 mV.^124^ Various studies have reported a range of diffusion coefficients for ATP in the cytoplasm. Effective diffusion coefficients as low as 100 μm^2^/s^125^ have been observed when accounting for path length and interactions with cytoplasmic components. Other reported values include 200 μm^2^/s,^126^ 236 μm^2^/s,^127^ and a temperature- and pH-dependent diffusion coefficient ranging from 153 μm^2^/s at pH 7.31 and 5°C to 480 μm^2^/s at pH 7.18 and 40°C.^128^ Furthermore, the diffusion coefficient is time-dependent; over long timescales, the translational diffusion of a molecule the size of ATP can be reduced to as little as 10–50% of its value in dilute solution.^129^ The average cytoplasmic microviscosity reported for HeLa cells is as high as 86 cP,^130^ nearly two orders of magnitude greater than the viscosity of water at 20 °C. According to the Stokes–Einstein relationship in the low Reynolds number regime ( $D=\frac{k_{B}T}{6\pi \eta r}$, where *D* is the diffusion coefficient, *k*_B_ is the Boltzmann constant, *η* is the viscosity, and *r* is the radius of the diffusing spherical particle), such high cytoplasmic microviscosity results in a diffusion coefficient that is two orders of magnitude lower than in water. Experimentally tracing all ATP molecules from their site of production to their site of consumption - anywhere within the cell - is not feasible. However, the above gathered evidence indicates that the ATP diffusion coefficient depends on multiple factors, including distance, time, microviscosity, molecular crowding, membrane exchange rates, pH, temperature, cell type, and more. These dependencies suggest that the diffusion coefficient can vary widely and may, under certain conditions, be significantly lower than commonly reported values. However, for our purposes, we adopt a value of 100 μm^2^/s.

##### Average ATP diffusion time

We investigate two modes of ATP transport: (1) diffusion, occurring in both prokaryotic and eukaryotic cells, and (2) mitochondrial motility, where ATP is delivered directly by mitochondria to various cellular compartments. In the case of free diffusion, the average diffusion time in three-dimensional space is estimated by $t\approx \frac{L^{2}}{6D}$, where *L* is the diffusion distance (approximated here as the cell length) and *D* is the diffusion coefficient. For cells with typical sizes - 1 μm for bacteria, 100 μm for ciliates,^43^ and 22 μm for adult human cells^36^ - the corresponding ATP diffusion times are approximately 0.002 s, 17 s, and 0.8 s, respectively. However, in the cytoplasm, ATP transport does not occur via free diffusion. Instead, it follows a drift-diffusion behavior, influenced by ATP concentration gradients across various organelles and compartments. ATP is actively targeted to specific sites based on metabolic demand. The average time required for ATP molecules to reach their target in 3D space under such conditions can be approximated by $t_{t}\approx \frac{L^{3}}{3Da}$, where *a* is the radius of the target region, assuming *L*≫*a*.^131^ Taking *a*=0.1*L*, the estimated ATP delivery times are approximately 0.03 s for bacteria, 330 s for ciliates, and 16 s for human cells. These back-of-the-envelope calculations illustrate the two extremes of ATP diffusion time across differently sized organisms, influencing the average ATP turnover time. In the second scenario, ATP is delivered by mitochondrial motility. Mitochondria are motile organelles with experimentally observed speeds ranging from 24.2 nm/s in dendrites to 185 nm/s in axons.^132^ Based on these velocities, ATP delivery times via mitochondria would be roughly 540–4132 s for a typical ciliate cell and 119–909 s for a typical adult human cell. This clearly shows that mitochondrial motility is not an efficient mechanism for rapid ATP delivery and that repositioning mitochondria within the cell is time-consuming. Taken together, this evidence supports the existence of ATP concentration gradients as a function of distance from mitochondria, varying with physiological conditions - a phenomenon well documented in the literature.^125^^,^^133^^,^^134^^,^^135^^,^^136^

In animals, ATP levels as low as 1.18 × 10^-4^ g ATP / g dry mass have been reported in amphipods in saltwater at 9^o^C,^60^ whereas values as high as 4.46 × 10^-1^ g ATP / g dry mass have been observed in the brain of nondiapausing pupae of *H. armigera*,^137^ illustrating a difference of more than three orders of magnitude. One of the main reasons for these differences is the mechanism by which oxygen is delivered to cells via closed, open, or mixed circulatory systems.^138^ In general, oxygen tension decreases with increasing distance from the vessel wall, as observed in various tissues across different organisms - for example, in the arterioles of the rat mesentery.^139^ A further reduction in oxygen availability can occur during intense activity; for example, in Weddell seals during diving, blood flow to organs such as the pancreas, liver, and heart decreases markedly,^140^ leading to reduced oxygen supply and consequently lower ATP levels. Other potential contributors to these differences include the broad range of cell sizes in animals and their varied metabolic activities, as discussed in the main text.

Both experimental and theoretical evidence suggest that oxygen molecules diffuse more rapidly in lipids than in cytosolic and interstitial fluids, due to their higher solubility in lipids, which helps overcome diffusion barriers over long distances.^141^ This suggests that in animal cells - similar to heterotrophic protists - some mitochondria should localize near the cell membrane, while others position themselves close to energy-demanding compartments such as the nucleus.^142^ Animals with high respiration rates, such as insects, tend to have more mitochondria than those with lower respiration rates, such as fish. This abundance enables greater flexibility in mitochondrial spatial distribution, allowing for more efficient positioning across the cell, thereby minimizing ATP delivery time and, consequently, ATP turnover time.

#### On the size-dependence of C:ATP

As shown in the compiled measurements of ATP content per unit biomass across the kingdoms of life, there is a statistically significant relationship between log_10_(organism or tissue dry mass) and the log_10_(C:ATP) ratio for several of the organisms and tissues examined, although some exhibit positive correlations while others show negative correlations. As discussed in the main text, we cannot fully elaborate on these patterns at this stage because we do not yet have an explicit model for size-dependent ATP turnover time. However, if we assume a constant ATP turnover time, we can plot our theoretically modeled log_10_(C:ATP) versus log_10_(dry mass) for the organisms studied here, where the positive or negative relationships arise simply from the scaling exponent of carbon content versus dry mass divided by the scaling exponent of respiration rate versus dry mass (Table S6). The result of this analysis is shown in Figure S6, which also illustrates how much variation in C:ATP is attributable to size differences across the observed size range for each organism.

We also performed a sensitivity analysis on the parameters in Equations 2 and 3 while keeping ATP turnover time constant. For each organism, we fixed all parameters at their baseline values. We then varied one parameter at a time across its prescribed plausible range (sampled on a uniform grid of 100 values) and recomputed the model-predicted C:ATP for each value. Sensitivity was summarized as a fold-change metric for each parameter, defined as “max(C:ATP)/min(C:ATP)” over that range. Figure S7 presents a two-dimensional heat map: one dimension shows variations in C:ATP caused by parameter changes within groups (row-wise comparison, blue map), and the other shows variations across groups (column-wise comparison, orange map). For example, to assess the importance of parameters influencing C:ATP in fish, one should examine the blue colors in the fish row. This indicates that $\frac{\text{ATP}}{O_{2}^{c}}$ has the strongest effect (darkest blue), whereas dry mass (DM) has the weakest effect (lightest blue). Likewise, to compare the effect of DM on C:ATP across groups, one should examine the orange colors in the DM column. This column shows that bacteria (darkest orange), followed by cyanobacteria, are the most strongly influenced by DM among the organisms.

### Quantification and statistical analysis

Python was used throughout this study to numerically solve the equations developed or compiled (Equations 1, 2, 3, and 4 in the main text and the equations presented in Tables S5 and S6), generate the figures (Figures 1 and S1–S7), produce Table 1, and perform the statistical analyses reported in Table S1. The statistical analyses include the sample size (*n*, number of C:ATP data points), coefficient of determination (R^2^), median values, regression slope of the log_10_(C:ATP) versus log_10_(tissue or cell dry weight), *p*-value, and 95% confidence intervals.

## Supplemental information

Document S1. Figures S1–S7 and Tables S1–S7

Data S1. Comprehensive ATP-content dataset spanning 397 measurements from 82 studies across the kingdoms of life (1964–2024)
