# Supplementary material for: Carbon-to-ATP ratios across the kingdoms of life
Source: iScience. 2026 Jul 29;29(8):116849. doi: 10.1016/j.isci.2026.116849 (PMC13451875; doi:10.1016/j.isci.2026.116849)
Supplement: Supplementary file 1 [file mmc1.pdf]

iScience, Volume *29*

Supplemental information

Carbon-to-ATP ratios across the kingdoms of life

Peyman Fahimi, Mohammad M. Amirian, Andrew J. Irwin, and Zoe V. Finkel

DATA S1

Comprehensive ATP-content dataset spanning 397 measurements from 82 studies across the kingdoms of life (1964–2024).

Mendeley Data Link*:*

https://data.mendeley.com/datasets/j4bwxwy6gz/1

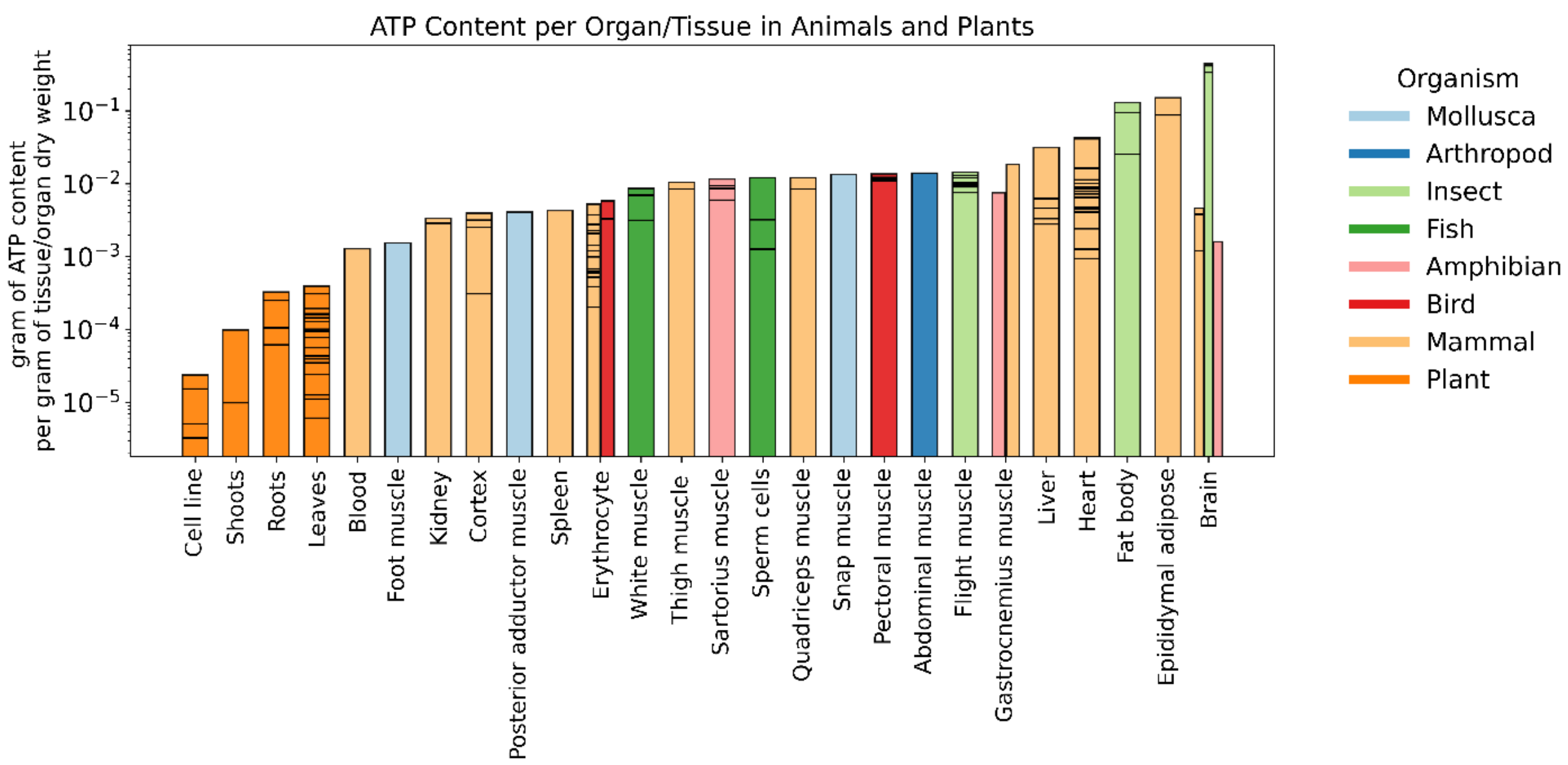

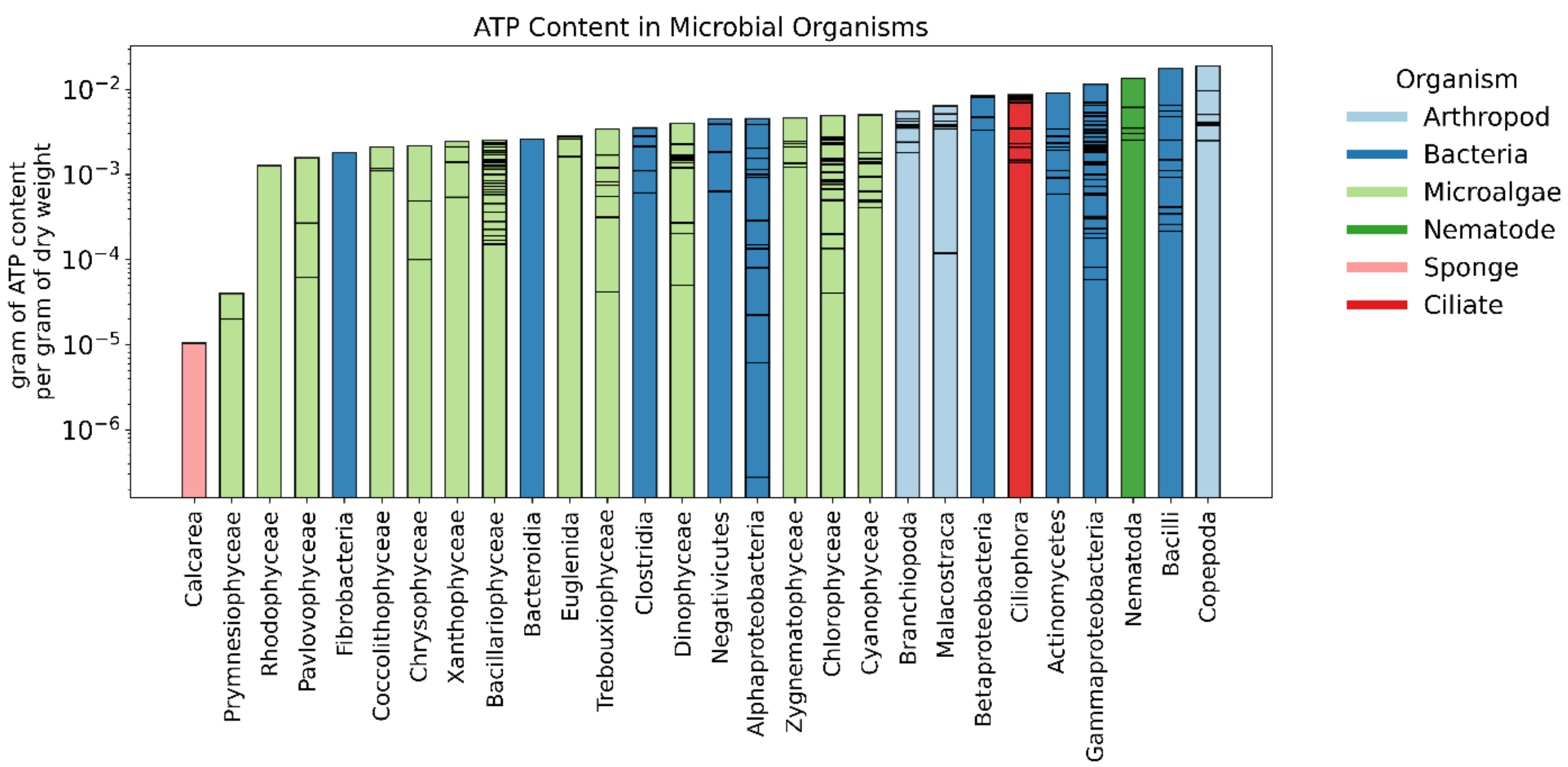

Figure S1: ATP content (g per g of dry mass) in various organs across different animal groups (top panel, n = 130 data points) and across diverse microbial organisms (bottom panel, n = 265 data points) under different physiological and environmental conditions. Each color represents a specific animal or microbial group, as indicated in the legends. Horizontal lines within the bars indicate ATP values of individual organisms within the same group.

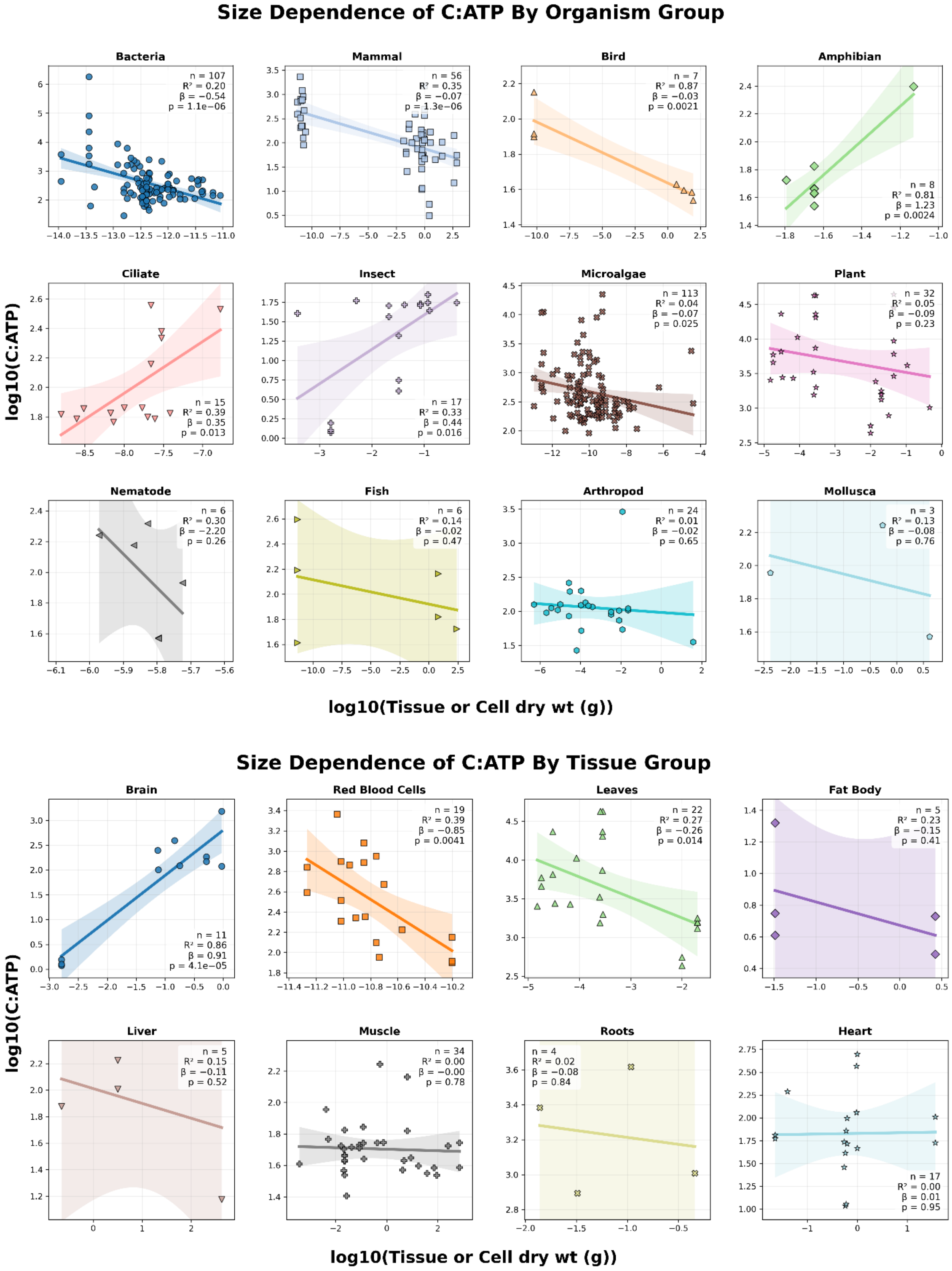

Figure S2: Size dependence of the C:ATP ratio across organisms (top panel) and tissues (bottom panel). Statistically significant relationships indicate a decrease in C:ATP with increasing mass in bacteria, microalgae, birds, mammals, erythrocytes (red blood cells), and leaves, and an increase in C:ATP with mass in insects, ciliates, amphibians, and brain tissue. Each subfigure reports the sample size (n), coefficient of determination (R^2), regression slope (β), and p-value; $p < 0.05$ is considered statistically significant. Subfigures are ordered by p-value, from most to least statistically significant. A detailed statistical summary is provided in Table S1.

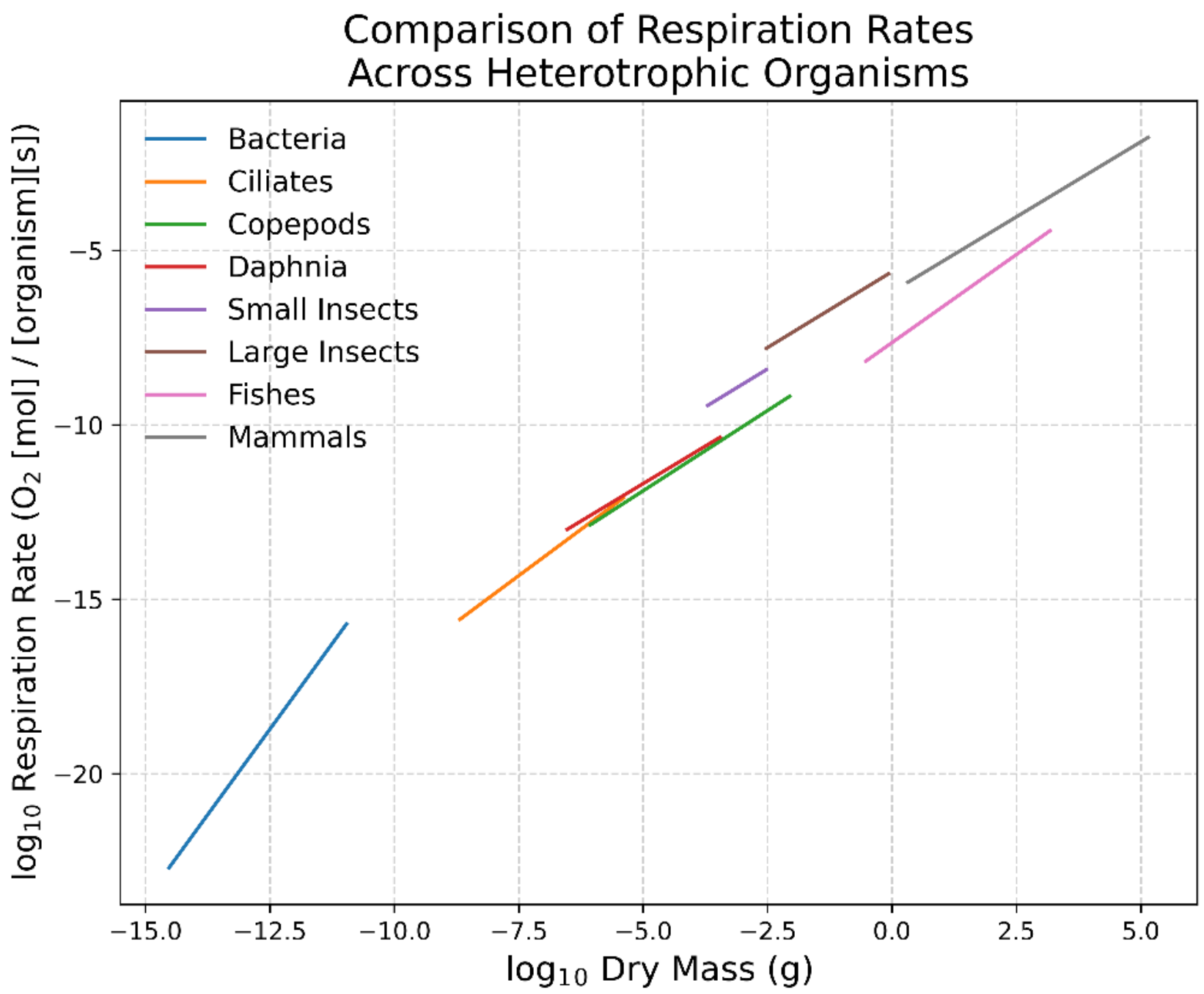

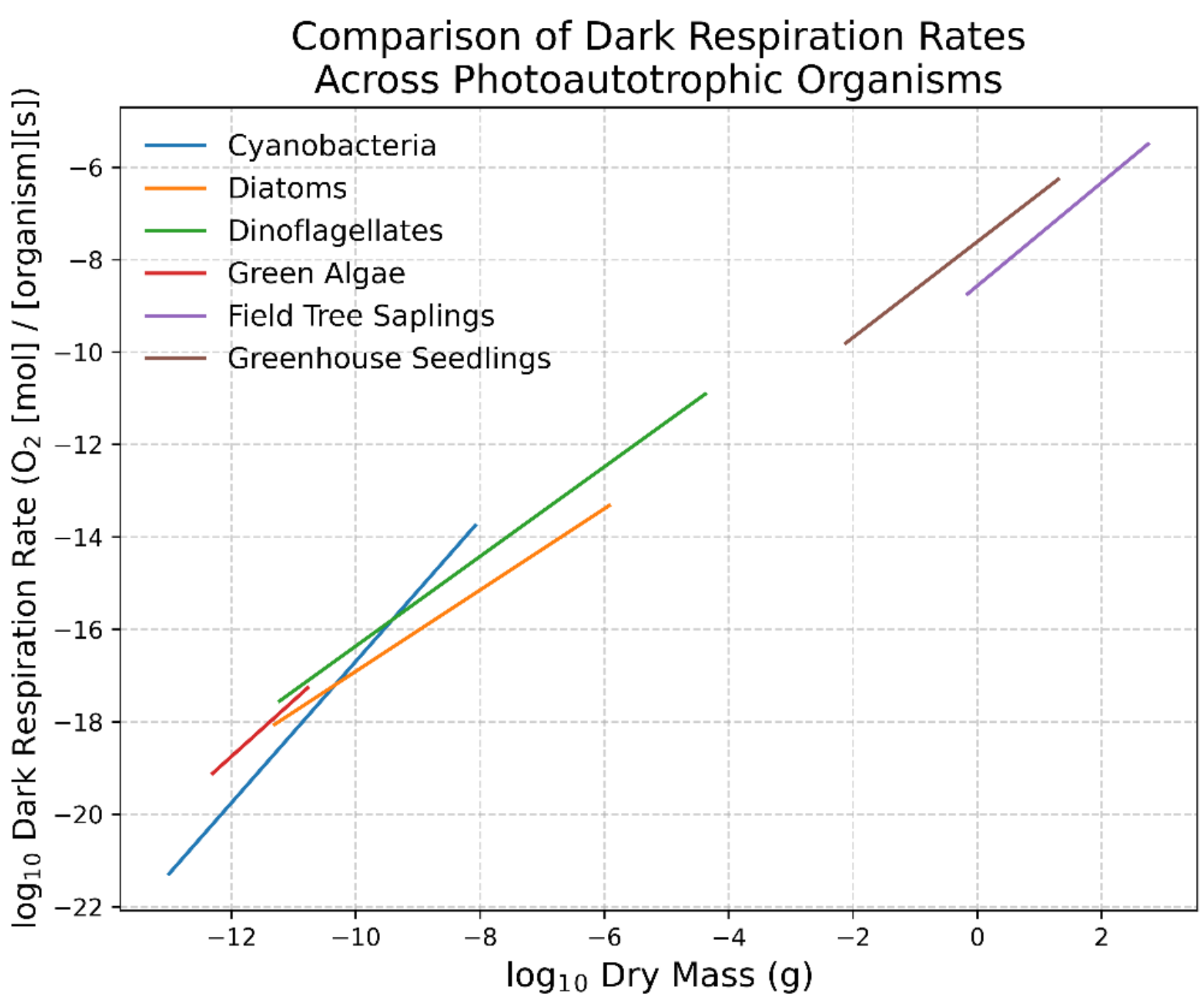

Figure S3: Respiration rate in heterotrophic (top panel) and photoautotrophic (bottom panel) organisms as a function of dry mass, ranging from bacteria to mammals and spanning 20 orders of magnitude. The respiration rates represent organisms in their active state.

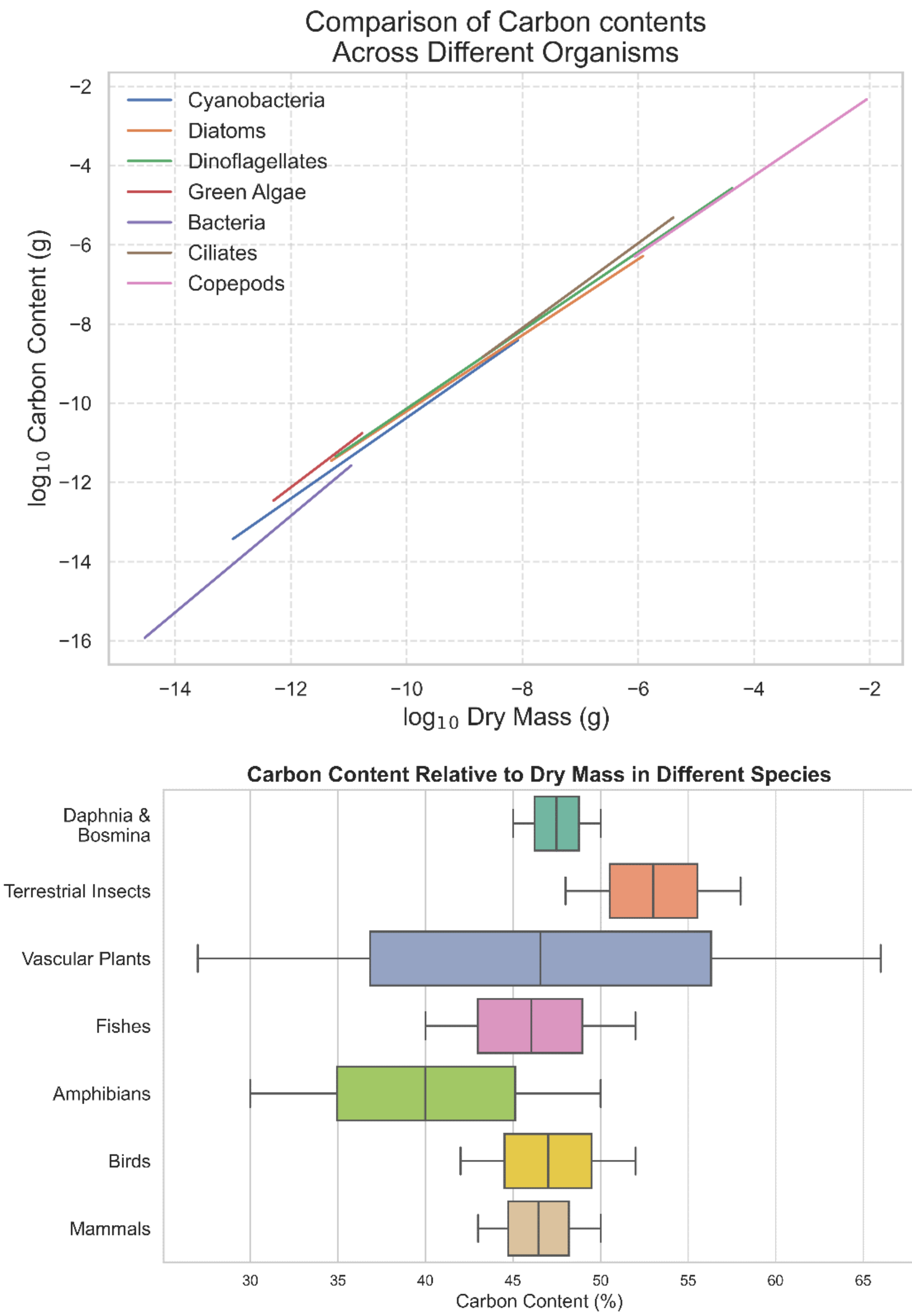

Figure S4: Carbon content versus dry mass relationship across different organisms (top and bottom panels).

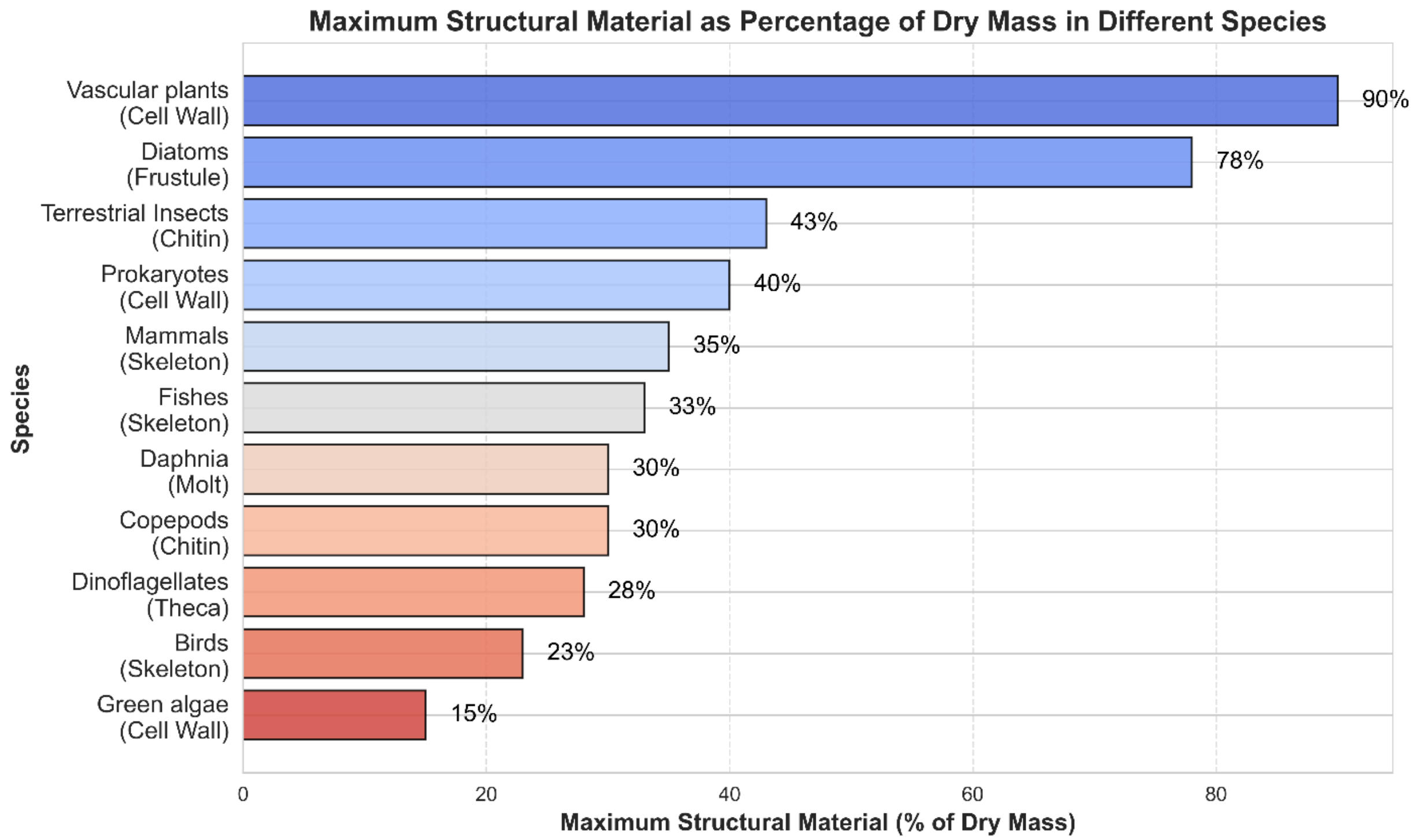

Figure S5: Percentage of maximum structural material relative to dry mass across different taxa.

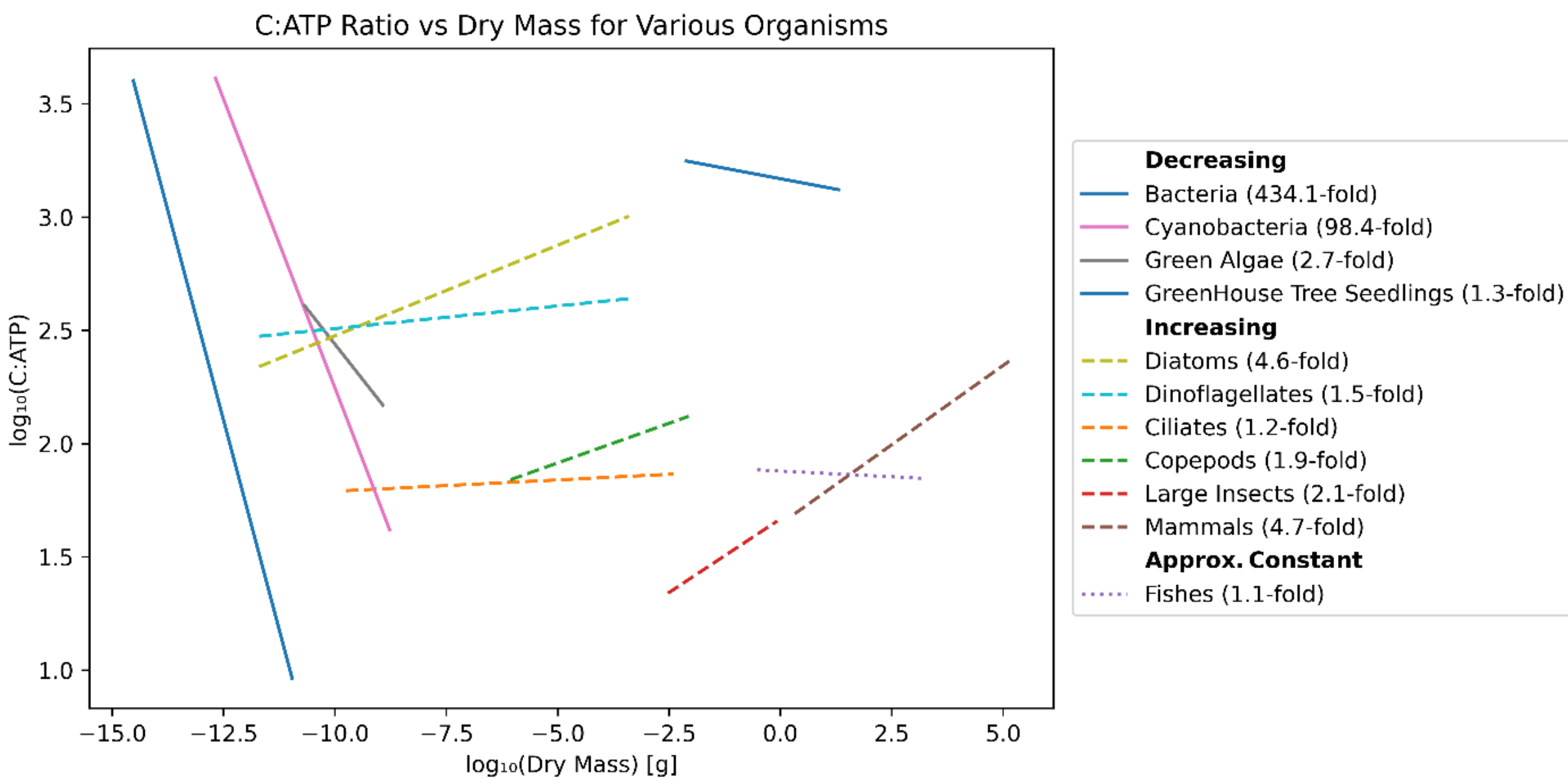

Figure S6: This figure shows the relationship between log(C:ATP) and log(Dry Mass [DM]) based on our model. In this analysis, only carbon content and respiration rate are modeled as functions of dry mass; all other parameters are fixed at their median values. The results indicate that in some organisms, C:ATP increases with DM, whereas in others it decreases or remains nearly constant. The legend reports the fold change in C:ATP across the full DM range, from smallest to largest sizes. Notably, bacteria and cyanobacteria show large variations because the exponent for respiration rate versus DM in these groups is much higher than that for carbon versus DM.

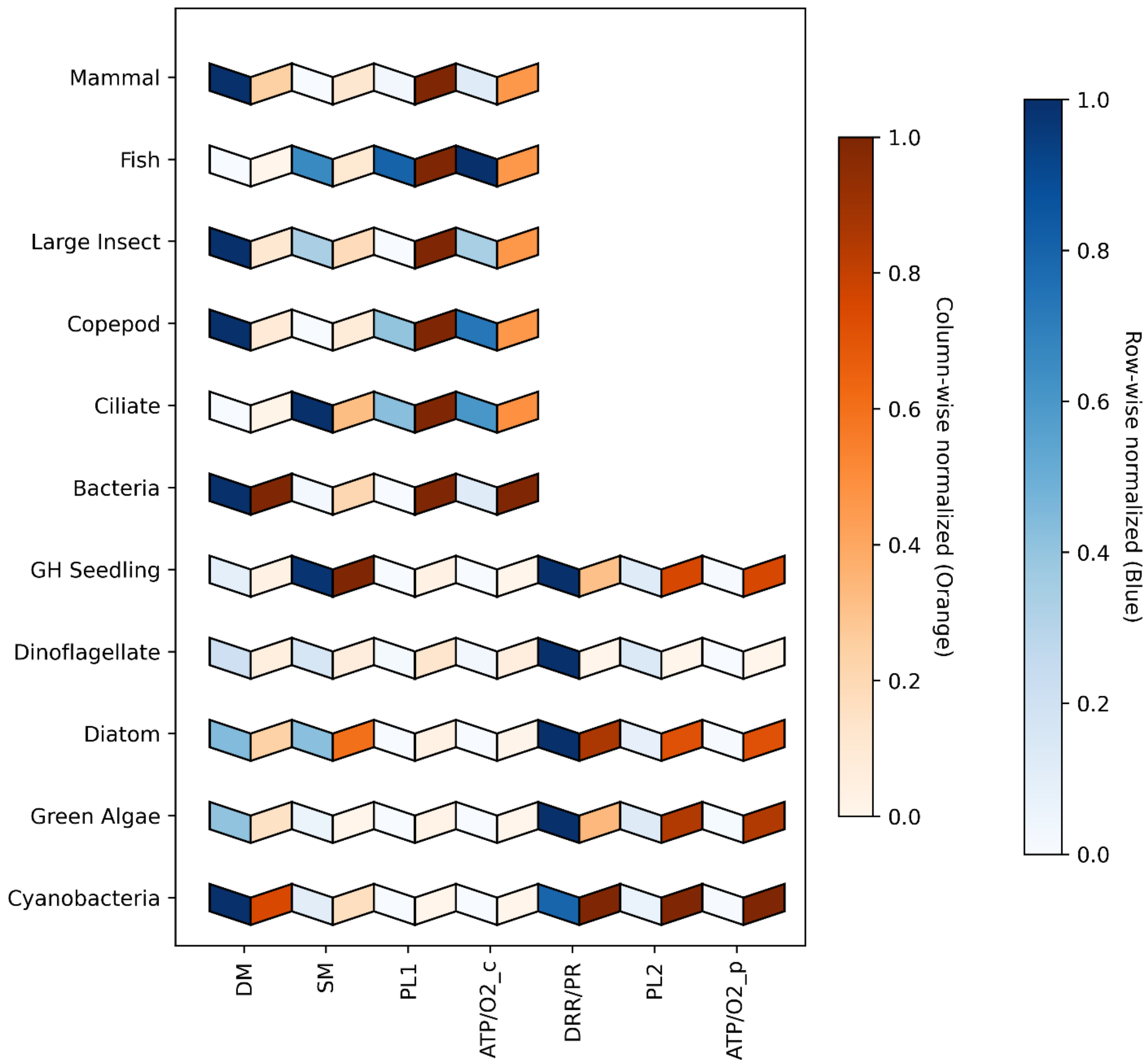

Figure S7: Two-dimensional sensitivity analysis of parameters in Eqs. (2) and (3) under the assumption of constant ATP turnover time. The heat map displays (i) within-group variation in C:ATP in response to parameter changes (row-wise, blue scale) and (ii) across-group variation for each parameter (column-wise, orange scale).

Table S1: Statistical summary of $\log_{10}$(C:ATP) versus $\log_{10}$(tissue or cell dry weight) across major organismal (top-panel) and tissue (bottom-panel) groups, ordered by p-value (from most to least significant). Reported metrics include the coefficient of determination (R^2), sample size (n), median C:ATP, regression slope (β), p-value, and 95% confidence interval (CI); $p < 0.05$ is considered statistically significant. The reported p-values and CIs correspond to the significance of the regression slope (β).

Organism Group	R^2	n	Median C:ATP	β	p-value	95% CI
**Bacteria**	0.20	108	255.7	–0.54 ± 0.10	1.08e–06	[–0.75, –0.33]
**Mammal**	0.35	56	120.9	–0.07 ± 0.01	1.27e–06	[–0.09, –0.04]
**Bird**	0.87	7	42.7	–0.03 ± 0.01	2.11e–03	[–0.05, –0.02]
**Amphibian**	0.81	8	46.0	1.23 ± 0.25	2.43e–03	[0.63, 1.84]
**Ciliate**	0.39	16	71.9	0.35 ± 0.12	1.29e–02	[0.09, 0.61]
**Insect**	0.33	17	43.8	0.44 ± 0.16	1.59e–02	[0.10, 0.79]
**Microalgae**	0.04	113	333.3	–0.07 ± 0.03	2.50e–02	[–0.13, –0.01]
**Plant**	0.05	32	3742.9	–0.09 ± 0.07	2.33e–01	[–0.24, 0.06]
**Nematode**	0.30	6	117.7	–2.20 ± 1.66	2.57e–01	[–6.81, 2.42]
**Fish**	0.14	6	105.8	–0.02 ± 0.02	4.74e–01	[–0.09, 0.05]
**Arthropod**	0.01	24	107.4	–0.02 ± 0.05	6.49e–01	[–0.12, 0.07]
**Mollusca**	0.13	3	90.3	–0.08 ± 0.20	7.62e–01	[–2.68, 2.52]

Tissue Group	R^2	n	Median C:ATP	β	p-value	95% CI
**Brain**	0.86	11	123.0	0.91 ± 0.12	4.14e–05	[0.63, 1.19]
**Red Blood Cells**	0.39	19	327.1	–0.85 ± 0.26	4.11e–03	[–1.38, –0.31]
**Leaves**	0.27	22	3050.5	–0.26 ± 0.10	1.36e–02	[–0.47, –0.06]
**Fat Body**	0.23	5	5.4	–0.15 ± 0.15	4.08e–01	[–0.64, 0.34]
**Liver**	0.15	5	101.5	–0.11 ± 0.15	5.20e–01	[–0.60, 0.38]
**Muscle**	0.00	34	48.4	0.00 ± 0.02	7.84e–01	[–0.04, 0.03]
**Roots**	0.02	4	1721.9	–0.08 ± 0.35	8.44e–01	[–1.60, 1.44]
**Heart**	0.00	17	59.1	0.01 ± 0.13	9.48e–01	[–0.27, 0.29]

Table S2: Range of dry mass for the studied organisms, extracted directly from the references or calculated assuming a dry mass-to-body mass ratio of 0.3 and a mass density similar to that of water.

Broad Groups		Phylum/Class /Family		Dry Mass Ranges in Grams [g]	Ref.
Phytoplankton		Diatoms		$2.0 \times 10^{-12} \leq DM \leq 4.0 \times 10^{-4}$<br>Median = 5.4×10^{-10}	1,2
		Dinoflagellates		$2.0 \times 10^{-12} \leq DM \leq 4.0 \times 10^{-4}$<br>Median = 3.9×10^{-10}	3
		Green Algae		$2.1 \times 10^{-11} \leq DM \leq 1.2 \times 10^{-9}$<br>Median = 1.1×10^{-10}	4
		Golden Algae		$1.3 \times 10^{-12} \leq DM \leq 1.0 \times 10^{-10}$<br>Mean = 10^{-11}	5,6
		Cyanobacteria		$2.1 \times 10^{-13} \leq DM \leq 1.7 \times 10^{-9}$<br>Median = 9.0×10^{-12}	7
Heterotrophic protists		Flagellates		$2.1 \times 10^{-12} \leq DM \leq 4.8 \times 10^{-6}$<br>$1.2 \times 10^{-9}$	8
		Ciliates		$1.8 \times 10^{-10} \leq DM \leq 4.0 \times 10^{-3}$<br>Median = 1.0×10^{-7}	9
Heterotrophic prokaryotes		Bacteria		$0.3 \times 10^{-14} \leq DM \leq 1.1 \times 10^{-11}$<br>Median = 2.1×10^{-13}	10
Multicellular Zooplankton		Tunicates		$3.5 \times 10^{-6} \leq DM \leq 1.3$<br>Median = 1.1×10^{-1}	11
		Copepods		$8.7 \times 10^{-7} \leq DM \leq 8.8 \times 10^{-3}$<br>Median = 1.9×10^{-4}	
		Euphausiids		$4.7 \times 10^{-6} \leq DM \leq 3.5 \times 10^{-1}$<br>Median = 1.58×10^{-2}	
		Amphipods		$6.0 \times 10^{-5} \leq DM \leq 0.1$<br>Median = 2.7×10^{-3}	
Freshwater Invertebrates		Cladocerans	*Daphnia*	$0.3 \times 10^{-6} \leq DM \leq 3.5 \times 10^{-4}$<br>Median = 1.4×10^{-5}	12,13
			Bosmina	$0.3 \times 10^{-6} \leq DM \leq 3.0 \times 10^{-6}$<br>Median = 1.4×10^{-6}	
Vascular Plants		Seedlings		$7.7 \times 10^{-3} \leq DM \leq 2 \times 10^{1}$<br>Median = 0.17	7
		Saplings		$0.7 \leq DM \leq 5.6 \times 10^{2}$<br>Median = 22	
Leaves		Lettuce<br>(*Lactuca sativa* L.)		$0.3 \leq DM \leq 1.6 \times 10^{1}$<br>Median = 5	14
Terrestrial Insects		Dictyoptera, Diptera, Homoptera, Hymenoptera, Lepidoptera, and Orthoptera		Small<br>$0.2 \times 10^{-3} \leq DM \leq 0.3 \times 10^{-2}$<br>Median = 0.6×10^{-3}<br>Large<br>$0.3 \times 10^{-2} \leq DM \leq 0.85$<br>Median = 0.12	15
Nematodes		---		$2.6 \times 10^{-8} \leq DM \leq 1.6 \times 10^{-6}$<br>Median = 3.8×10^{-7}	16
Vertebrates	Ectotherms	Fishes		$0.3 \leq DM \leq 1.5 \times 10^{3}$<br>Median = 109.2	17
		Amphibians		$0.3 \leq DM \leq 1.5 \times 10^{2}$<br>Median = 1.95	
	Endotherms	Birds		$0.9 \leq DM \leq 7.8 \times 10^{2}$<br>Median = 8.1	
		Mammals		$2.1 \leq DM \leq 1.4 \times 10^{5}$<br>Median = 61.5	

Table S3: Mass scaling of respiration rates across the kingdoms of life. For details on how these equations were derived, see METHOD DETAILS II.4 in the STAR METHODS.

Broad Groups	Phylum/Class /Family	Units	Relation Between Respiration Rate (*RR*) or Dark Respiration Rate (*DRR*) and Carbon (*C*), Body Mass (*BM*), Dry Mass (*DM*), or Volume (*V*)	Temp. ºC	Ref.
Phytoplankton	All	$DRR\left[\frac{\text{pmol O}_2}{\text{cell h}}\right]$<br>$V[\mu m^3]$<br>$C[\text{pg}]$<br>$DM[\text{pg}]$	$DRR = 7.94\times10^{-5}\,V^{0.9}$<br>$DRR = 3.98\times10^{-4}\,C^{1.02}$<br>$DRR = 1.38\times10^{-4}\,DM^{0.98}$	18	18
	Diatoms	$DRR\left[\frac{\text{mg }C}{\text{cell h}}\right]$<br>$C[\text{pg}]$<br>$DM[\text{pg}]$<br>$V[\mu m^3]$	$DRR = 1.68\times10^{-11}\,C^{0.80}$<br>$DRR = 3.32\times10^{-12}\,V^{0.77}$<br>$DRR = 5.33\times10^{-12}\,DM^{0.84}$	20	19
		$DRR\left[\frac{\text{W}}{\text{cell}}\right]$<br>$BM[\text{pg}]$	$DRR = 3.30\times10^{-14}\,BM^{0.88}$<br>$DRR = 9.54\times10^{-14}\,DM^{0.88}$	25	7
	Dinoflagellates	$DRR\left[\frac{\text{pl O}_2}{\text{cell h}}\right]$<br>$C[\text{pg}]$<br>$DM[\text{pg}]$	$DRR = 0.026\,C^{1.01}$<br>$DRR = 0.04\,DM^{0.97}$	25	20
	Green Algae		$DRR = 0.008\,C^{1.21}$<br>$DRR = 0.001\,DM^{1.43}$		
	Golden Algae		$DRR = 0.03\,C^{0.99}$<br>$DRR = 0.05\,DM^{0.95}$		
	Cyanobacteria	$RR\left[\frac{\text{W}}{\text{cell}}\right]$<br>$BM[\text{pg}]$	$DRR = 1.36\times10^{-15}\,BM^{1.53}$<br>$DRR = 8.56\times10^{-15}\,DM^{1.53}$	25	7
Heterotrophic protists	Ciliates, Flagellates, and Amoeba	$RR\left[\frac{\text{W}}{\text{cell}}\right]$<br>1 ml O_2 ~ 20 j<br>$BM[\text{g}]$	$RR = 10^{-1.46}(BM)^{1.06}$<br>$RR = 0.12(DM)^{1.06}$	20	10,21
Heterotrophic prokaryotes	Bacteria		$RR = 10^{10.2}(BM)^{1.96}$<br>$RR = 1.68\times10^{11}(DM)^{1.96}$		
Multicellular Zooplankton	All	$RR\left[\frac{\mu\text{l O}_2}{\text{ind. h}}\right]$<br>$DM\left[\frac{\text{mg}}{\text{ind.}}\right]$	$RR = 2.65(DM)^{0.79}$<br>$RR = 7.60(C)^{0.83}$	25	22
	Tunicates	$RR\left[\frac{\mu\text{l O}_2}{\text{ind. h}}\right]$<br>$C\left[\frac{\mu\text{g}}{\text{ind.}}\right]$	$RR = 0.017\,C^{0.93}$<br>$RR = 0.002\,DM^{0.87}$	15	23
	Copepods		$RR = 0.008\,C^{0.93}$<br>$RR = 0.005\,DM^{0.92}$		
	Euphausiids		$RR = 0.04\,C^{0.77}$<br>$RR = 0.024\,DM^{0.75}$		
	Amphipods		$RR = 0.009\,C^{0.94}$<br>$RR = 0.004\,DM^{0.94}$		
Freshwater Invertebrates	Cladocerans – *Daphnia*	$RR\left[\frac{\mu\text{l O}_2}{\text{ind.d}}\right]$<br>$DM[\mu\text{g}]$	$RR = 0.35(DM)^{0.86}$	20	12
	Cladocerans – *Bosmina*		$RR = 0.31(DM)^{0.94}$		
Vascular Plants<br><br>Field, Greenhouse (GH), and Growth-Chamber (GC) Plants	Field Tree Saplings	$DRR\left[\frac{\text{nmol O}_2}{\text{ind. s}}\right]$<br>$DM[\text{g}]$	$DRR = 2.46(DM)^{1.117}$	24	24
	GH Tree Seedlings		$DRR = 22.13(DM)^{1.037}$		
	GC Tree Seedlings		$DRR = 29.65(DM)^{1.058}$		
Leaves	Lettuce<br>(*Lactuca sativa* L.)	$DRR\left[\frac{\mu\text{mol O}_2}{\text{ind. s}}\right]$	$DRR = 0.275(1-\exp(-0.119DM))$	25	14

			$DM[\text{g}]$			
Terrestrial Insects		Dictyoptera, Diptera, Homoptera, Hymenoptera, Lepidoptera, and Orthoptera	$RR\left[\frac{\text{mm}^3\ \text{O}_2}{\text{ind. h}}\right]$ $BM[\text{mg}]$	Large: $RR = 129.94(BM)^{0.87}$ $RR = 370.38(DM)^{0.87}$ Small: $RR = 31.62(BM)^{0.87}$ $RR = 90.13(DM)^{0.87}$	22	[15]
Nematodes		Note: Only species occurring under oxic conditions were included.	$RR\left[\frac{\text{nmol O}_2}{\text{ind. d}}\right]$ $DM[\mu\text{g}]$	$RR = 0.79(DM)^{1.07}$	10	[16]
Vertebrates	Ectotherms	Fishes	$RR\left[\frac{\text{ml O}_2}{\text{ind. h}}\right]$ $BM[\text{g}]$	$RR = 0.55(BM)^{1.01}$ $RR = 1.84(DM)^{1.01}$	25	[17]
		Amphibians		$RR = 0.54(BM)^{1.08}$ $RR = 1.98(DM)^{1.08}$		
	Endotherms	Birds		$RR = 21.11(BM)^{0.85}$ $RR = 58.44(DM)^{0.85}$	38	
		Mammals		$RR = 18.69(BM)^{0.86}$ $RR = 52.45(DM)^{0.86}$		

Table S4: Mass scaling of carbon content across the kingdoms of life. For details on how these equations were derived, see METHOD DETAILS II.4 in the STAR METHODS.

**Broad Groups**	**Phylum/Class /Family**	**Units**	**Relationship Between Carbon Content (*C*) and Dry Mass (*DM*), Volume (*V*), or Wet Mass (*WM*)**	**Ref.**
Phytoplankton	Diatoms	$C\left[\frac{pg}{cell}\right]$ $DM\left[\frac{pg}{cell}\right]$ $V[\mu m^3]$	$C = 1.21(V)^{0.75}$ $C = 0.75(DM)^{0.96}$	25 26
	Dinoflagellates		$C = 1.20(V)^{0.94}$ $C = 0.77(DM)^{0.99}$	
	Green Algae		$C = 0.094(V)^{1.088}$ $C = 0.18(DM)^{1.18}$	
	Golden Algae		$C = 0.02(V)^{1.218}$ $C = 0.04(DM)^{1.32}$	26
	Cyanobacteria		$C = 0.22(V)^{0.939}$ $C = 0.39(DM)^{1.02}$	
Heterotrophic protists	Loricate Ciliates		$C = 0.68(V)^{0.841}$ $C = 1.14(DM)^{0.91}$	
	Aloricate Ciliates		$C = 0.23(V)^{0.984}$ $C = 0.42(DM)^{1.07}$	
	Heterotrophic Dinoflagellates		$C = 0.28(V)^{0.900}$ $C = 0.48(DM)^{0.98}$	
Heterotrophic prokaryotes	Bacteria	$C\left[\frac{fg}{cell}\right]$ $DM\left[\frac{pg}{cell}\right]$	$C = 72.24(V)^{1.12}$ $C = 143.2(DM)^{1.22}$	27
Multicellular Zooplankton	Tunicates	$C\left[\frac{mg}{ind.}\right]$ $WM\left[\frac{mg}{ind.}\right]$ $DM\left[\frac{mg}{ind.}\right]$	$C = 0.002(WM)^{1.01}$ $C = 0.76(DM)^{0.93}$	11
	Copepods		$C = 0.12(WM)^{0.95}$ $C = 0.55(DM)^{0.99}$	
	Euphausiids		$C = 0.09(WM)^{1.01}$ $C = 0.46(DM)^{0.98}$	
	Amphipods		$C = 0.10(WM)^{0.92}$ $C = 0.38(DM)^{1}$	
Freshwater Invertebrates	Cladocerans		$0.45\ DM \leq C \leq 0.50\ DM$	28,29
Plants – Reproductive organs	---		$0.31\ \mathrm{DM} \leq C \leq 0.60\ DM$	30
Plants – Leaves			$0.27\ \mathrm{DM} \leq C \leq 0.66\ DM$	
Plants – Stems			$0.30\ \mathrm{DM} \leq C \leq 0.66\ DM$	
Plants – Roots			$0.27\ \mathrm{DM} \leq C \leq 0.64\ DM$	
Terrestrial Insects	Orthoptera & Coleoptera		$0.48\ \mathrm{DM} \leq C \leq 0.58\ DM$	31,32
Nematodes	---		$C \sim 0.525\ DM$	33
Vertebrates	Fishes		$0.40\ \mathrm{DM} \leq C \leq 0.52\ DM$	34
	Amphibians		$0.30\ \mathrm{DM} \leq C \leq 0.50\ DM$	35
	Birds		$0.42\ \mathrm{DM} \leq C \leq 0.52\ DM$	
	Mammals		$0.43\ \mathrm{DM} \leq C \leq 0.50\ DM$	

Table S5: Structural material-to-dry mass ratios across the kingdoms of life. When structural material mass is described as ranging from zero to a specific value, it indicates that the structural material cannot be definitively excluded, as it may be metabolically active. Therefore, a range of values starting from zero to a maximum is considered in the numerical simulation of the carbon-to-ATP ratio.

**Broad Groups**	**Phylum/Class /Family**	**Structural Material**	**Relationship Between Structural Material (*SM*) and Dry Mass**	**Ref.**
Phytoplankton	Diatoms	Frustule	$0 \leq SM \leq 0.78DM$	36
	Dinoflagellates	Theca	$0 \leq SM \leq 0.37\,C$<br>$0 \leq SM \leq 0.28\,DM$	26
	Green Algae	Cell wall	$0 \leq SM \leq 0.15DM$	37
	Haptophytes	Coccolith	$SM[\text{pg}] = 2.7k_s V[\mu m^3]$,<br>$0.01 \leq k_s \leq 0.2$, median $k_s = 0.05$,<br>$SM[\text{pg}] = 0.25(DM[\text{pg}])^{1.09}$,	38
	Golden Algae	Siliceous scale	$0 \leq SM \leq 0.78DM$	36,39
	Cyanobacteria	Cell wall	$0 \leq SM \leq 0.40DM$	40
Heterotrophic protists	Loricate Ciliates	Lorica	$0 \leq SM \leq 0.55DM$	41
	Heterotrophic Dinoflagellates	Theca	$0 \leq SM \leq 0.37C$<br>$0 \leq SM \leq 0.28\,DM$	26
Heterotrophic prokaryotes	Bacteria	Cell wall	$0 \leq SM \leq 0.40DM$	40
Multicellular Zooplankton	Tunicates	Tunic	$0 \leq SM \leq 0.50DM$	42
	Copepods<br>Euphausiids<br>Amphipods	Chitin	$0 \leq SM \leq 0.30DM$	43
Freshwater Invertebrates	Cladocerans	Molt	$SM[\mu\text{g}] = 0.93(L[\text{mm}])^{3.23}$<br>$DM[\text{mg}] = 0.055L[\text{mm}] - 0.052$	12<br>44
Vascular Plants	---	Cell wall	$0 \leq SM \leq 0.9DM$	---
Terrestrial Insects	Orthoptera & Coleoptera	Chitin	$0 \leq SM \leq 0.43DM$	45
Nematodes	---	---	$SM \sim 0$	---
Vertebrates	Fishes	Skeleton	$0 \leq SM \leq 0.10BM$<br>$0 \leq SM \leq 0.33DM$	46
	Amphibians	Skeleton	$0 \leq SM \leq 0.2BM$<br>$0 \leq SM \leq 0.66DM$	---
	Birds	Skeleton	$SM[\text{kg}] = 0.065(BM[\text{kg}])^{1.071}$<br>$SM[\text{g}] = 0.14(DM[\text{g}])^{1.071}$	47
	Mammals	Skeleton	$SM[\text{kg}] = 0.061(BM[\text{kg}])^{1.090}$<br>$SM[\text{g}] = 0.12(DM[\text{g}])^{1.090}$	

Table S6: Respiration rates standardized to $\left[\frac{mol\ O_2}{organism\ s}\right]$ *at 25°C, except for mammals and birds, for which respiration rates are reported at their typical physiological body temperatures. Carbon contents and dry mass are expressed in grams.*

**Broad Groups**	**Phylum/Class /Family**	**Carbon Content in [g] versus Dry Mass in [g]**	**Respiration Rates Standardized to** $\left[\frac{\text{mol O}_2}{\text{org. s}}\right]$ **at 25°C versus Dry mass in [g]**
Phytoplankton	All	---	$DRR = 4.19 \times 10^{-8}\ DM^{0.98}$
	Diatoms	$C = 0.25DM^{0.96}$	$DRR = 7.73 \times 10^{-9}\ DM^{0.88}$
	Dinoflagellates	$C = 0.58DM^{0.99}$	$DRR = 2.16 \times 10^{-7}\ DM^{0.97}$
	Green Algae	$C = 28.39DM^{1.18}$	$DRR = 1.73 \times 10^{-3}\ DM^{1.43}$
	Golden Algae	$C = 276.7DM^{1.32}$	$DRR = 1.56 \times 10^{-7} DM^{0.95}$
	Cyanobacteria	$C = 0.68DM^{1.02}$	$DRR = 0.04\ DM^{1.53}$
Heterotrophic protists	Ciliates	$C = 2.9DM^{1.07}$	$RR = 4.23 \times 10^{-7} DM^{1.06}$
	Flagellates	$C = 0.27DM^{0.98}$	
Heterotrophic prokaryotes	Bacteria	$C = 62.51DM^{1.22}$	$RR = 5.93 \times 10^{5} DM^{1.96}$
Multicellular Zooplankton	All		$RR = 7.7 \times 10^{-9} DM^{0.79}$
	Tunicates	$C = 0.47DM^{0.93}$	$RR = 1.03 \times 10^{-8} DM^{0.87}$
	Copepods	$C = 0.51DM^{0.99}$	$RR = 5.13 \times 10^{-8} DM^{0.92}$
	Euphausiids	$C = 0.40DM^{0.98}$	$RR = 2.35 \times 10^{-8} DM^{0.75}$
	Amphipods	$C = 0.38DM^{1}$	$RR = 5.41 \times 10^{-8} DM^{0.94}$
Freshwater Invertebrates	Cladocerans – *Daphnia*	$0.45\ DM \leq C \leq 0.50\ DM$	$RR = 4.13 \times 10^{-8} DM^{0.86}$
	Cladocerans – *Bosmina*		$RR = 1.1 \times 10^{-7} DM^{0.94}$
Vascular Plants<br>Field, Greenhouse (GH), and Growth-Chamber (GC) Plants	Field Tree Saplings	$0.27\ \text{DM} \leq C \leq 0.66\ DM$	$DRR = 2.69 \times 10^{-9} DM^{1.117}$
	GH Tree Seedlings		$DRR = 24.25 \times 10^{-9} DM^{1.037}$
	GC Tree Seedlings		$DRR = 32.49 \times 10^{-9} DM^{1.058}$
Leaves	Lettuce (*Lactuca sativa* L.)		$DRR = 0.275 \times 10^{-6}\left(1 - e^{(-0.119DM)}\right)$
Terrestrial Insects	Dictyoptera, Diptera, Homoptera, Hymenoptera, Lepidoptera, and Orthoptera	$0.48\ \text{DM} \leq C \leq 0.58\ DM$	Large:<br>$RR = 2.46 \times 10^{-6} DM^{0.87}$<br>Small:<br>$RR = 5.99 \times 10^{-7} DM^{0.87}$
Nematodes	---	$C \sim 0.525\ DM$	$RR = 9.52 \times 10^{-8} DM^{1.07}$
Vertebrates – Ectotherms	Fishes	$0.40\ \text{DM} \leq C \leq 0.52\ DM$	$RR = 2.28 \times 10^{-8} DM^{1.01}$
	Amphibians	$0.30\ \text{DM} \leq C \leq 0.50\ DM$	$RR = 2.46 \times 10^{-8} DM^{1.08}$
Vertebrates – Endotherms	Birds	$0.42\ \text{DM} \leq C \leq 0.52\ DM$	$RR = 7.25 \times 10^{-7} DM^{0.85}$
	Mammals	$0.43\ \text{DM} \leq C \leq 0.50\ DM$	$RR = 6.50 \times 10^{-7} DM^{0.86}$

Table S7: Minimum and maximum ATP yield per oxygen molecule consumed in oxidative phosphorylation and aerobic glycolysis, associated with the number of c-subunits in ATP synthase and the metabolic pathway.

Number of c-subunits	Considering H^+ associated with PiC?	Minimum $\frac{ATP_{yield}}{O_{2-c-max}} \rightarrow$ succinate to malate pathway	Maximum $\frac{ATP_{yield}}{O_{2-c-max}} \rightarrow$ glycogen to CO_2 pathway
8	Yes	3.27	5.74
9	No	4	6.83
10	Yes	2.77	4.99
	No	3.6	6.23
11	No	3.27	5.74
12		3	5.33
13		2.77	4.99
14		2.57	4.7
15		2.4	4.43
16		2.25	4.21
17		2.12	4.01

References

1. Hasle, G.R., Syvertsen, E.E., Steidinger, K.A., Tangen, K., and Tomas, C.R. (1996). Identifying marine diatoms and dinoflagellates (Elsevier).
2. Snoeijs, P., Busse, S., and Potapova, M. (2002). The importance of diatom cell size in community analysis. J. Phycol. *38*, 265–281.
3. Hoppenrath, M. (2017). Dinoflagellate taxonomy—a review and proposal of a revised classification. Mar. Biodivers. *47*, 381–403.
4. Nielsen, S.L. (2006). Size-dependent growth rates in eukaryotic and prokaryotic algae exemplified by green algae and cyanobacteria: comparisons between unicells and colonial growth forms. J. Plankton Res. *28*, 489–498.
5. Graham, L.E., and Wilcox, L.W. (2000). Algae (Prentice-Hall).
6. Siver, P.A. (2022). The downsizing of gigantic scales and large cells in the genus Mallomonas (*Synurales*, *Chrysophyceae*). Sci. Rep. *12*, 4896.
7. Makarieva, A.M., Gorshkov, V.G., Li, B.-L., Chown, S.L., Reich, P.B., and Gavrilov, V.M. (2008). Mean mass-specific metabolic rates are strikingly similar across life's major domains: evidence for life's metabolic optimum. Proc. Natl. Acad. Sci. USA. *105*, 16994–16999.
8. Lisicki, M., Velho Rodrigues, M.F., Goldstein, R.E., and Lauga, E. (2019). Swimming eukaryotic microorganisms exhibit a universal speed distribution. eLife *8*, e44907.
9. Corliss, J.O. (2016). The ciliated protozoa: characterization, classification and guide to the literature (Pergamon Press).
10. DeLong, J.P., Okie, J.G., Moses, M.E., Sibly, R.M., and Brown, J.H. (2010). Shifts in metabolic scaling, production, and efficiency across major evolutionary transitions of life. Proc. Natl. Acad. Sci. USA. *107*, 12941–12945.
11. Kiørboe, T. (2013). Zooplankton body composition. Limnol. Oceanogr. *58*, 1843–1850.
12. Lynch, M. (2024). The bioenergetic cost of building a metazoan. Proc. Natl. Acad. Sci. USA. *121*, e2414742121.
13. Lynch, M., Trickovic, B., and Kempes, C.P. (2022). Evolutionary scaling of maximum growth rate with organism size. Sci. Rep. *12*, 22586.
14. Van Iersel, M.W. (2003). Carbon use efficiency depends on growth respiration, maintenance respiration, and relative growth rate. A case study with lettuce. Plant Cell Environ. *26*, 1441–1449.
15. Niven, J.E., and Scharlemann, J.P.W. (2005). Do insect metabolic rates at rest and during flight scale with body mass? Biol. Lett. *1*, 346–349.
16. Maciute, A., Holovachov, O., Berg, P., Glud, R.N., Broman, E., Nascimento, F.J.A., and Bonaglia, S. (2021). A microsensor-based method for measuring respiration of individual nematodes. Methods Ecol. Evol. *12*, 1841–1847.
17. Gillooly, J.F., Gomez, J.P., and Mavrodiev, E. V. (2017). A broad-scale comparison of aerobic activity levels in vertebrates: endotherms versus ectotherms. Proc. Roy. Soc. B *284*, 20162328.
18. López-Sandoval, D.C., Rodríguez-Ramos, T., Cermeño, P., Sobrino, C., and Marañón, E. (2014). Photosynthesis and respiration in marine phytoplankton: Relationship with cell size, taxonomic affiliation, and growth phase. J. Exp. Mar. Biol. Ecol. *457*, 151–159.
19. Finkel, Z.V. (1998). Diatoms: size and metabolic processes (MSc Thesis, Dalhousie University).
20. Tang, E.P.Y. (1995). The allometry of algal growth and respiration (MSc Thesis, McGill University).
21. Okie, J.G. (2012). Microorganisms. In: Metabolic ecology: a scaling approach (WILEY-BLACKWELL), 133–153.
22. Ikeda, T. (1985). Metabolic rates of epipelagic marine zooplankton as a function of body mass and temperature. Mar. Biol. *85*, 1–11.
23. Kiørboe, T., and Hirst, A.G. (2014). Shifts in mass scaling of respiration, feeding, and growth rates across life-form transitions in marine pelagic organisms. Am. Nat. *183*, E118–E130.
24. Reich, P.B., Tjoelker, M.G., Machado, J.L., and Oleksyn, J. (2006). Universal scaling of respiratory metabolism, size and nitrogen in plants. Nature *439*, 457–461.
25. Finkel, Z. V., Follows, M.J., and Irwin, A.J. (2016). Size-scaling of macromolecules and chemical energy content in the eukaryotic microalgae. J. Plankton Res. *38*, 1151–1162.
26. Menden-Deuer, S., and Lessard, E.J. (2000). Carbon to volume relationships for dinoflagellates, diatoms, and other protist plankton. Limnol. Oceanogr. *45*, 569–579.
27. Fagerbakke, K.M., Heldal, M., and Norland, S. (1996). Content of carbon, nitrogen, oxygen, sulfur and phosphorus in native aquatic and cultured bacteria. Aquat. Microb. Ecol. *10*, 15–27.
28. Frost, P.C., Xenopoulos, M.A., and Larson, J.H. (2004). The stoichiometry of dissolved organic carbon, nitrogen, and phosphorus release by a planktonic grazer, *Daphnia*. Limnol. Oceanogr. *49*, 1802–1808.

29. Walve, J., and Larsson, U. (1999). Carbon, nitrogen and phosphorus stoichiometry of crustacean zooplankton in the Baltic Sea: implications for nutrient recycling. J. Plankton Res. *21*, 2309–2321.
30. Ma, S., He, F., Tian, D., Zou, D., Yan, Z., Yang, Y., Zhou, T., Huang, K., Shen, H., and Fang, J. (2018). Variations and determinants of carbon content in plants: a global synthesis. Biogeosciences *15*, 693–702.
31. Bertram, S.M., Bowen, M., Kyle, M., and Schade, J.D. (2008). Extensive natural intraspecific variation in stoichiometric (C:N:P) composition in two terrestrial insect species. J. Insect Sci. *8*, 26.
32. Trakimas, G., Krams, R., Krama, T., Kortet, R., Haque, S., Luoto, S., Eichler Inwood, S., Butler, D.M., Jõers, P., Hawlena, D., et al. (2019). Ecological stoichiometry: a link between developmental speed and physiological stress in an omnivorous insect. Front. Behav. Neurosci. *13*, 42.
33. Persson, T. (1983). Influence of soil animals on nitrogen mineralisation in a northern Scots pine forest. In: New Trends Soil Biol., Proc. 8th Int. Colloq. Soil Zool. pp. 117–126.
34. Hendrixson, H.A., Sterner, R.W., and Kay, A.D. (2007). Elemental stoichiometry of freshwater fishes in relation to phylogeny, allometry and ecology. J. Fish Biol. *70*, 121–140.
35. May, E.M., and El-Sabaawi, R.W. (2022). Life stage and taxonomy the most important factors determining vertebrate stoichiometry: a meta-analysis. Ecol. Evol. *12*, e9354.
36. Sicko-Goad, L.M., Schelske, C.L., and Stoermer, E.F. (1984). Estimation of intracellular carbon and silica content of diatoms from natural assemblages using morphometric techniques. Limnol. Oceanogr. *29*, 1170–1178.
37. Northcote, D.H., Goulding, K.J., and Horne, R.W. (1958). The chemical composition and structure of the cell wall of *Chlorella pyrenoidosa*. Biochem. J. *70*, 391.
38. Young, J.R., and Ziveri, P. (2000). Calculation of coccolith volume and its use in calibration of carbonate flux estimates. Deep. Sea. Res. 2. Top. Stud. Oceanogr. *47*, 1679–1700.
39. Sandgren, C.D., Hall, S.A., and Barlow, S.B. (1996). Siliceous scale production in chrysophyte and synurophyte algae. I. Effects of silica-limited growth on cell silica content, scale morphology, and the construction of the scale layer of *Synura petersenii*. J. Phycol. *32*, 675–692.
40. Doetsch, R.N., and Cook, T.M. (1973). Introduction to bacteria and their ecobiology (Springer Nature).
41. Gilron, G.L., and Lynn, D.H. (1989). Assuming a 50% cell occupancy of the lorica overestimates *tintinnine* ciliate biomass. Mar. Biol. *103*, 413–416.
42. Dunlop, M.J., Clemons, C., Reiner, R., Sabo, R., Agarwal, U.P., Bissessur, R., Sojoudiasli, H., Carreau, P.J., and Acharya, B. (2020). Towards the scalable isolation of cellulose nanocrystals from tunicates. Sci. Rep. *10*, 19090.
43. Tang, W.J., Fernandez, J.G., Sohn, J.J., and Amemiya, C.T. (2015). Chitin is endogenously produced in vertebrates. Curr. Biol. *25*, p897–900.
44. Ku, D., Chae, Y.J., Choi, Y., Ji, C.W., Park, Y.S., Kwak, I.S., Kim, Y.J., Chang, K.H., and Oh, H.J. (2022). Optimal method for biomass estimation in a cladoceran species, *daphnia magna* (straus, 1820): evaluating length–weight regression equations and deriving estimation equations using body length, width and lateral area. Sustainability *14*, 9216.
45. Lease, H.M., and Wolf, B.O. (2010). Exoskeletal chitin scales isometrically with body size in terrestrial insects. J. Morphol. *271*, 759–768.
46. Berrios-Lopez, M., Lewis, A.R., and Hensley, D.A. (1996). Scaling of skeletal mass to body mass in fishes. J. Morphol. *227*, 87–92.
47. Prange, H.D., Anderson, J.F., and Rahn, H. (1979). Scaling of skeletal mass to body mass in birds and mammals. Am. Nat. *113*, 103–122.
